# Prostate Cancer: A Review of Genetics, Current Biomarkers and Personalised Treatments

**DOI:** 10.1002/cnr2.70016

**Published:** 2024-10-16

**Authors:** Trevor K. Wilson, Oliver T. Zishiri

**Affiliations:** ^1^ Discipline of Genetics, School of Life Sciences, College of Agriculture, Engineering, and Science University of KwaZulu‐Natal Durban South Africa

**Keywords:** biomarkers, genes, personalised treatment, prostate cancer

## Abstract

**Background:**

Prostate cancer is the second leading cause of cancer deaths in men, second only to lung cancer. Despite this, diagnosis and prognosis methods remain limited, with effective treatments being few and far between. Traditionally, prostate cancer is initially tested for through a prostate serum antigen (PSA) test and a digital rectum examination (DRE), followed by confirmation through an invasive prostate biopsy. The DRE and biopsy are uncomfortable for the patient, so less invasive, accurate diagnostic tools are needed. Current diagnostic tools, along with genes that hold possible biomarker uses in diagnosis, prognosis and indications for personalised treatment plans, were reviewed in this article.

**Recent Findings:**

Several genes from multiple families have been identified as possible biomarkers for disease, including those from the MYC and ETS families, as well as several tumour suppressor genes, Androgen Receptor signalling genes and DNA repair genes. There have also been advances in diagnostic tools, including MRI‐targeted and liquid biopsies. Several personalised treatments have been developed over the years, including those that target metabolism‐driven prostate cancer or those that target inflammation‐driven cancer.

**Conclusion:**

Several advances have been made in prostate cancer diagnosis and treatment, but the disease still grows year by year, leading to more and more deaths annually. This calls for even more research into this disease, allowing for better diagnosis and treatment methods and a better chance of patient survival.

## 1 Introduction

With around 1.5 million cases [1, 2], prostate cancer ranks near the top of the world's cancer list. It is very prevalent in developed countries, but outside of the developed world, Southern Africa's prostate cancer rate of 65.9 in 100 000 is the highest, with a mortality rate of 22 in 100 000 [1]. The reason for this higher mortality in Southern Africa is suspected to be due to men from this region being less willing to seek medical help, possibly due to financial reasons [3]. This statistic is quite concerning, with more and more men succumbing to this awful disease. However, the key to successful treatment of many cancers, including prostate cancer, is early detection and screening, due to this cancer's symptoms only becoming distinguishable when the stage for curing the disease has passed [4]. Therefore, the best hope for many at‐risk individuals is for screening for early stages of prostate cancer development. If the disease is diagnosed in the early stages, biomarker testing can be performed to determine the best course of treatment to be used, and personalised medicine can be administered to each patient, allowing for better outcomes in each case [5]. However, many developing countries lack access to personalised medicines due to a lack of funding, facilities, expertise and so on [6]. Therefore, the development of biomarkers associated with genetic mutations resulting in prostate cancer must be undertaken to allow for more accessible personalised medicine to be developed and used for better outcomes. This review will focus on the genetics of prostate cancer, along with current trends in biomarker development and personalised treatments.

## 2 Background on Prostate Cancer

### 2.1 Cancer of the Prostate

**FIGURE 1:**
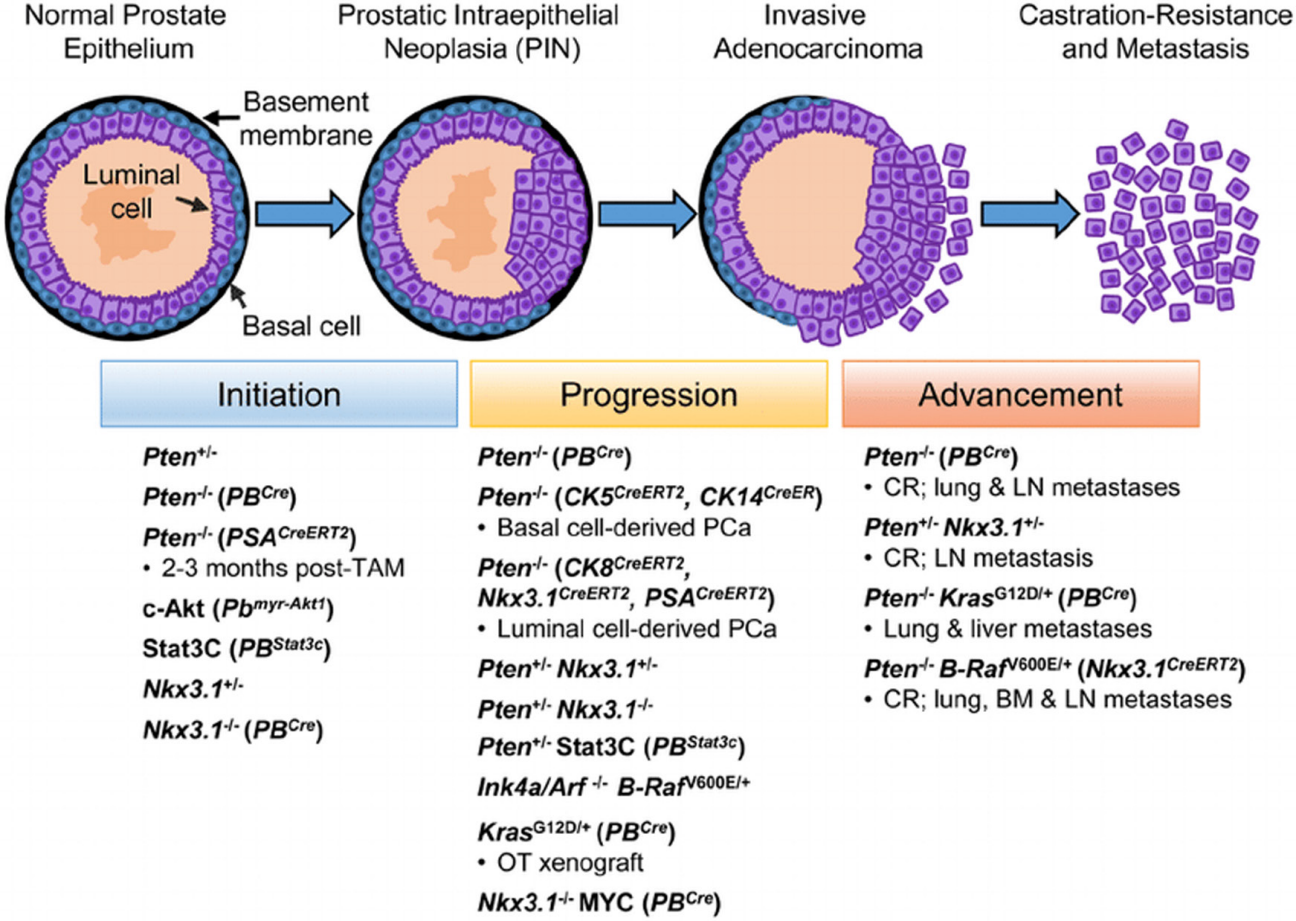
Process of malignant transformation within prostate cells. The figure is taken from Rybak, Bristow and Kapoor [10] (Open License CC BY).

As the name suggests, this cancer of the prostate can occur in all three regions of the prostate but is most associated with the peripheral zone, with up to 75% of all prostate cancers originating from this region [7]. For prostate cancer to occur, the prostate undergoes malignant transformation, where the cells within the prostate become cancerous following multiple steps, starting with a PIN (prostatic intraepithelial neoplasia), resulting in localised prostate cancer, bringing about prostate adenocarcinoma in the advanced stages and eventual metastasis [8, 9] (Figure 1).

Genetic mutations are the leading cause of prostate cancer [11]. These mutations in healthy prostate cells cause either changes in gene regulation or loss of function of the gene, resulting in uncontrolled growth of these cells, leading to the development of prostatic intraepithelial neoplasia (PIN), which is considered to be the first stepping stone toward prostate cancer [12]. Further alteration to the genetic makeup of these prostate cells results in a carcinoma, which is cancerous tissue arising from epithelial cells. The final advancement in prostate cancer is metastasis, the spreading of the cancer to other regions of the body [12].

### 2.2 Epidemiology of Prostate Cancer

The Global Cancer Observatory (GLOBOCAN) released global cancer statistics most recently in 2020, which will be used to assess the incidence, mortality and survival rates, along with the trends shown over the years by this disease [13]. The following few sections explain these trends in more detail.

#### 2.2.1 The Prevalence of Prostate Cancer

**FIGURE 2:**
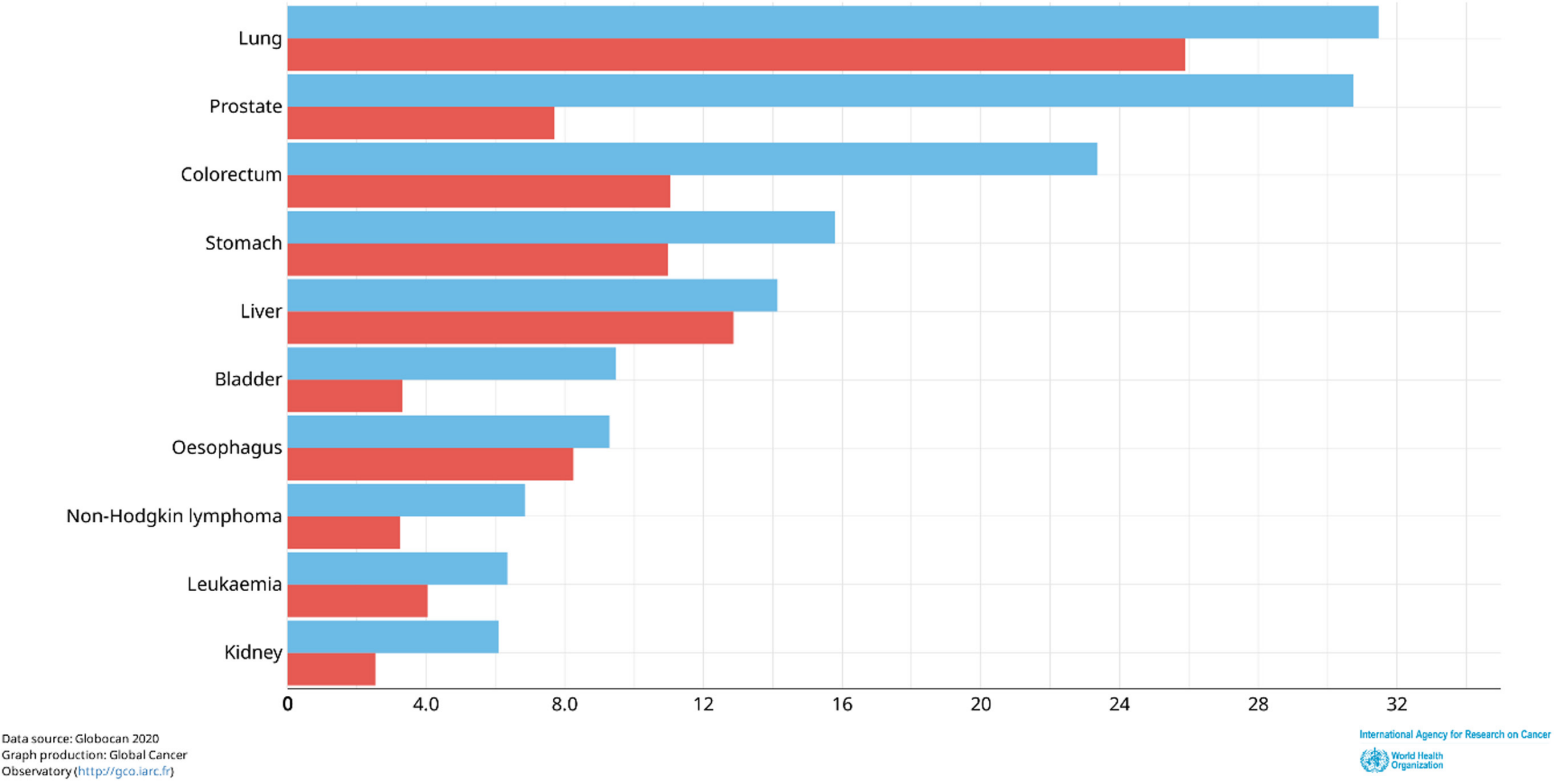
Mortality (red) and Age Standardised Incidence (blue) rates of common cancers in men, 2020. As can be seen, prostate cancer is the second leading cancer in terms of incidence, behind lung cancer, while being the sixth highest cancer in terms of mortality, falling behind lung, colorectum, stomach, liver and oesophageal cancer. *Image Source:* [13].

**FIGURE 3:**
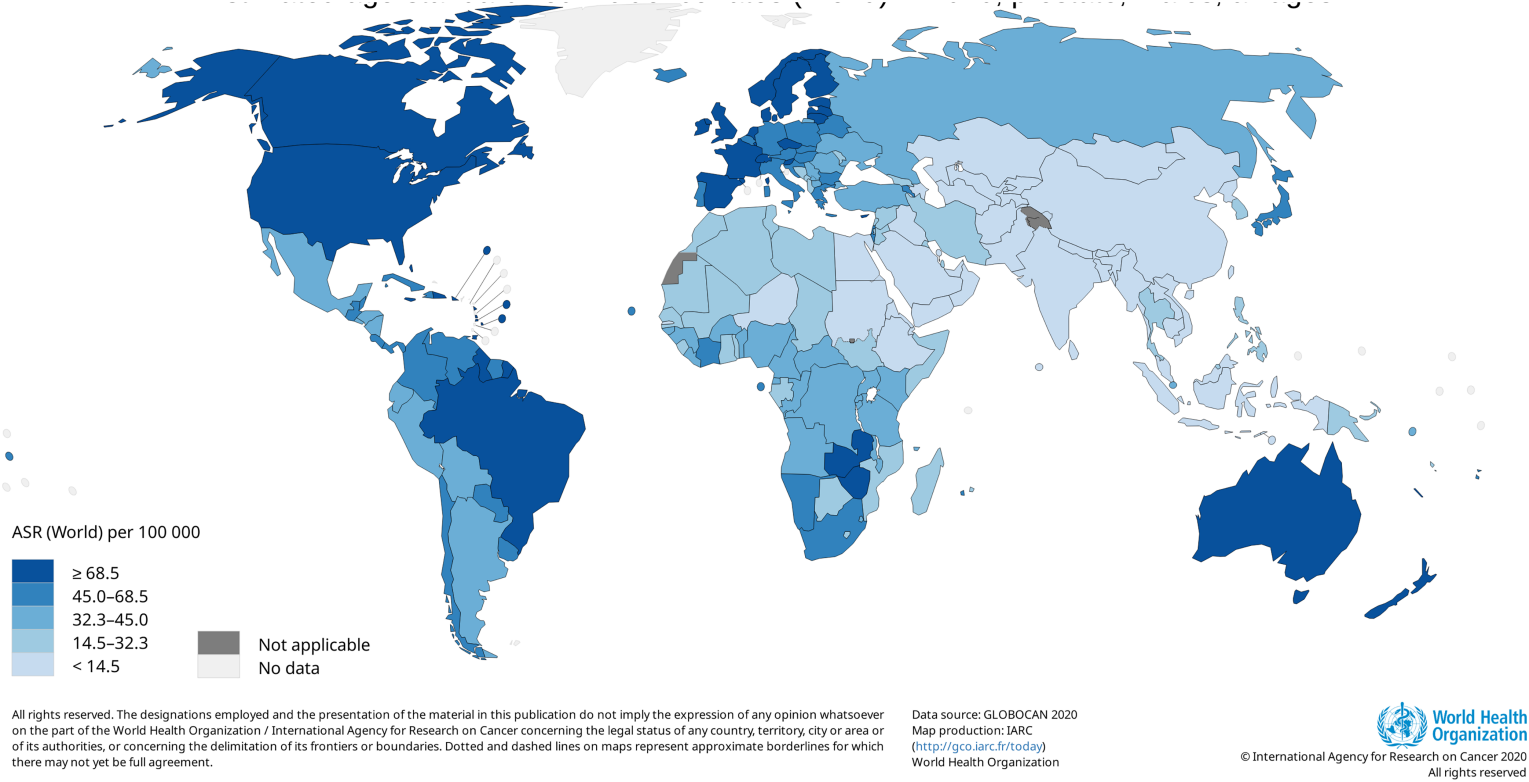
Map showing the worldwide ASR incidence rates for Prostate cancer based on 2020 statistics. *Image Source:* [13]. More developed countries have higher incidence rates than less developed countries, most likely due to more testing being conducted in these developed countries, leading to more cases being identified.

Prostate cancer is the second most prevalent cancer in men after lung cancer (Figure 2) [13]. This is per the trends from the 2018 estimates, just with more significant incidence rates now being recorded [14]. The year 2020 recorded more than 1 400 000 new prostate cancer cases, making up 15.14% of all new cases recorded that year, more than double the percentage recorded 2 years prior. Age‐standardised incidence rates (ASRs) were highest in North America (73 per 100 000) and lowest in Asia (13.6) (Table 1) [13]. This difference in incidence rates is significant but is likely due to the differences in diagnostic testing, with North American healthcare systems providing more thorough diagnostic testing than Asian systems [2]. However, the risk of this malignancy increases as the individual ages. Therefore, looking at the crude incidence rates worldwide, for men under the age of 50, the incidence rate is below 1 per 100 000 and increases to 98.3 for men between 50 and 59, while men aged 60 and above have an incidence rate of 508.8 [13]. It is worth noting that the African continent has the second‐lowest ASR incident rate but the highest mortality rate among all continental populations, which is of high concern. A map of the worldwide incidence rates can be seen below (Figure 3).

**TABLE 1:** New prostate cancer cases, age‐standardised incidence and mortality rates for the different continental regions based on GLOBOCAN data [13].

Population	Number	ASR incidence rate	ASR mortality rate
Northern America	239 574	73	8.3
Oceania	22 421	70.3	11
Europe	473 344	63.4	11.1
Latin America and the Caribbean	214 522	59.2	14.2
Africa	93 173	29.7	16.3
Asia	371 225	13.6	4.4

*Note:* The data indicates that the highest incidence rates come from North America, Oceania and Europe, while the lowest comes from Latin America, Africa and Asia. However, the highest mortality rates come from Latin America and Africa despite the lower incidence rates.

#### 2.2.2 Mortality

**FIGURE 4:**
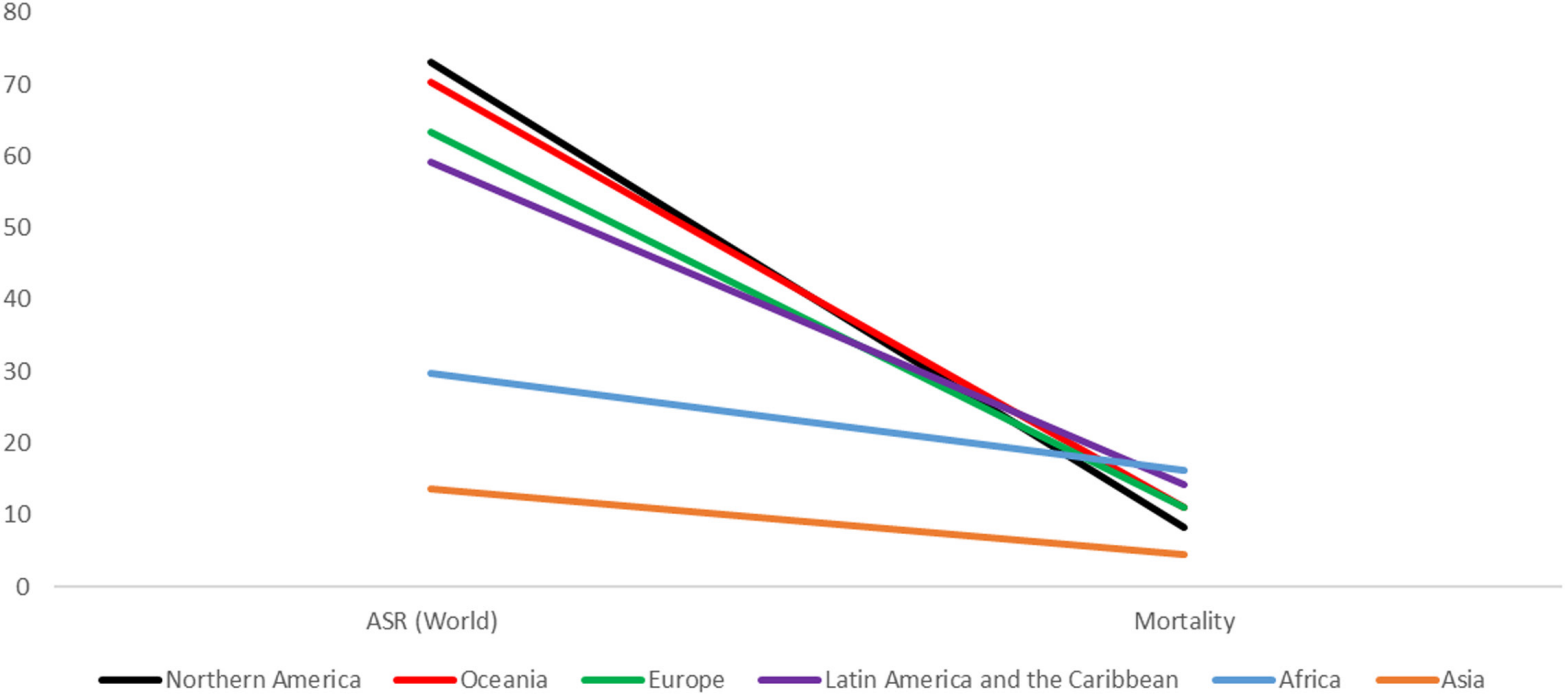
Graph showing the inverse relationship between prostate cancer mortality and incidence rates among continental populations. The regions with higher incidence rates tend to have a lower mortality rate. Data based on GLOBOCAN [13] statistics.

Mortality rates, like the incidence rates, vary significantly between continental populations but are almost inversely proportional to the incidence rates. As previously alluded to, the highest incidence rate belongs to Northern America. However, this population also has the second lowest mortality rate, meaning that North America has the most significant number of prostate cancer cases but one of the fewest numbers of deaths, likely due to a much higher level of health care compared to those continents with higher mortality rates. While Africa may have the second lowest incidence rate, it does hold the spot for having the highest mortality rate. This inverse proportionality can be seen in all continents except Asia, where incidence and mortality rates are low (Figure 4). This inverse relationship is, again, due to the significantly higher diagnostic testing rates in the more developed continental populations, which, while resulting in a higher incidence rate, allows for earlier detection of disease and better treatment outcomes, resulting in a lowered mortality rate [2]. Another reason for the discrepancy between incidence and mortality rates could be the healthcare infrastructure within these continents. As previously alluded to, continents like North America, Europe and Asia have significantly better healthcare systems and infrastructure in place, allowing for better and more effective treatment, resulting in fewer mortalities in these regions, while continents such as Africa and South America have less advanced and fewer healthcare systems in place, resulting in fewer diagnoses and more deaths to this disease.

**FIGURE 5:**
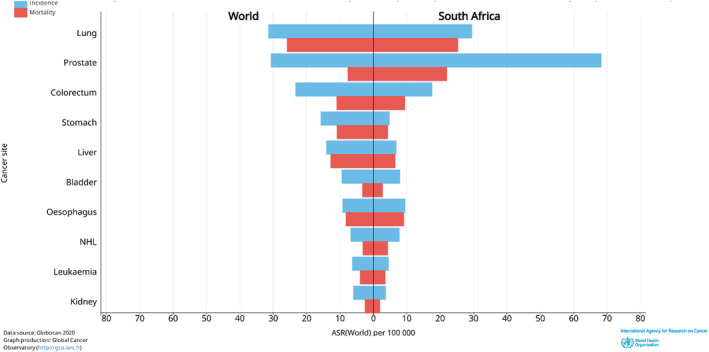
ASR incidence (blue) and Mortality (red) rates of the world's average and South Africa's population. *Image Source:* [13].

Comparing the world's average incidence and mortality rates to those of South Africa, a significant difference can be witnessed, with South Africa having a much greater rate in both options (Figure 5). This demonstrates that, although South Africa has an excellent diagnostic rate for the disease, allowing for earlier detection, the treatment options available are still not meeting the mark to meet the world average. Comparing the results from this figure, we can see a much closer relationship between South Africa and the world average in terms of all other cancers listed, with only prostate cancer being the outlier concerning mortality. This shows the need for improved treatment options in the country to help improve the outcomes of patients in need.

### 2.3 Signs and Symptoms of Prostate Cancer

Many men affected by Prostate cancer may be asymptomatic until it is too late for effective treatment, so regular medical check‐ups, such as PSA tests and prostate exams, are essential as one's age increases [15]. However, the most common symptoms experienced by men with prostate cancer are lower urinary tract symptoms (LUTSs). LUTSs can roughly be separated into three different classes: storage (frequency, urgency, nocturia, etc.), voiding (hesitancy, poor‐intermittent stream, dribbling, etc.) and post‐micturition (incomplete emptying). The commonality of LUTSs increases with age, but although an association has been found with localised prostate cancer, its association with advanced forms of the disease has not yet been identified [16, 17]. Because of this, patients with LUTSs could have a more significant likelihood of early‐stage prostate cancer, which could result in better treatment outcomes for those patients diagnosed with the disease from LUTSs.

However, it is important to note that LUTSs and any other signs of prostate cancer do not explicitly confirm disease presence. There is no symptom or sign found in prostate cancer patients that is found to be solely associated with the disease, with almost all symptoms being commonly associated with patients suffering from an enlarged prostate due to benign prostatic hyperplasia (BPH) or prostatitis, resulting in an inflamed prostate, which can cause pain when urinating [18].

### 2.4 Risk Factors

Risk factors can be anything that could increase the likelihood of an individual developing a disease or illness, such as cancer [19]. In prostate cancer, there are several different risk factors, many of which are unavoidable factors, such as age and ethnicity. In contrast, other factors can be altered to lower disease risk, such as diet and smoking. These risk factors will be described below.

#### 2.4.1 Age

By far, the risk factor most associated with this form of cancer is increasing age, and for good reason. As previously alluded to, a man's probability of prostate cancer development increases drastically with age, with men under 50 years of age having an incidence rate of less than 1 per 100 000 while being closer to 100 between 51 and 60 years of age, and increasing even further to over 500 per 100 000 in men over the age of 60 [13]. As with all cancers, age plays a major role in cancer onset. The development of cancer is a complex process with many factors that can take several years to pan out fully [20]. As we age, so do our cells, which become more susceptible to damage from both internal and external factors, which leads to more chances of genetic mutations resulting in cancer [21, 22].

#### 2.4.2 Ethnicity

Although not as strongly linked with increased incidence as increasing age, ethnicity does affect the chances of developing prostate cancer, with African men being at higher risk than men of other ethnicities for developing the disease [23, 24]. However, understanding of this disease is scarce among men living in sub‐Saharan Africa, even with this knowledge of the increased risk for African men, and so incidence rates for these populations are being underestimated due to this lack of knowledge, minimal screening and the shortage of healthcare access [24, 25]. Two studies conducted independently from one another identified a region on chromosome 8q24 that was significantly associated with an increased risk of prostate cancer, more so in African men than any other ethnicity [26, 27]. An SNP on the gene *CYP17* (rs743572) has been identified to create an increased risk of prostate cancer in African men [28].

A study out of the UK assessed over 700 000 men in primary care, looking for trends in PSA levels in men of different ethnicities [29]. The results from this study showed that African men tended to have higher PSA levels than any other ethnicity, especially in men over the age of 60. Furthermore, the study indicated that African men had higher incidence rates of prostate cancer in the year following the elevated PSA levels than any other ethnicity. The study also showed that Asian men had the lowest PSA levels and diagnoses of all ethnicities.

Koga, Song [30] conducted a study to determine which genomic alterations were more associated with men of African descent than men of other ethnicities. The results showed that African men had higher rates of mutations in certain genes, such as higher frequencies of *ZFHX3* gene mutations, *ETV3* deletions and *MYC* amplifications, than other ethnic groups. This study also showed less frequent *PTEN* and *TMPRSS2* mutations in African men. Further genes such as *PALB2*, *BRCA2* and *ATM* were identified as having elevated frequencies in prostate cancer in African men, while genes *NCOA2*, *PCAT1* and *STK19* showed more significant numbers of mutations in African men [31, 32].

#### 2.4.3 Obesity

An often‐avoidable risk factor for many men found with this disease is obesity. Previous studies into this cancer have found that obese individuals have a greater probability of advanced prostate cancer along with having worse prognoses than non‐obese men [33]. Recently, Vidal et al. [34] explained the increased mortality rate in obese men over non‐obese men. This same study also investigated whether the outcome of the disease differed in obese men of different races. The results indicated that a combination of both ethnicity and obesity risk factors did not heighten the cancer mortality risk. However, it was found that obesity is linked to the next factor below, which is diet. A person with a high body mass index (BMI, kg/m^2^) is at higher risk of lethal prostate cancer than an individual with a low BMI [35, 36]. Along with this, an individual who is less physically active is also at higher risk [37].

#### 2.4.4 Diet

Dietary patterns can affect the risk levels of an individual regarding prostate cancer. Shin et al. [38] recently compared three different dietary patterns: Traditional, Western and Prudent. The traditional diet followed the diet characteristic of the Japanese culture, which included high volumes of chicken, seafood, pickles and sake. In contrast, the prudent diet consists of vegetables, fish, fruits and legumes, and the Western diet is very red meat and high fat focused. From this study, the outcomes indicated that individuals with a Western dietary pattern tend to be at greater risk of disease development. At the same time, a prudent diet reduces the risk of disease. Similar studies have shown the same outcome between these two diets, showing that a high‐fat Western diet puts an individual at higher risk for prostate cancer [39, 40, 41]. Richman et al. [42] found that choline intake was associated with increased lethal prostate cancer risk. Egg yolks are high in choline, suggesting a diet containing many eggs could lead to a more lethal prostate cancer. Other foods associated with increased risk of this disease include processed red meats [43], dairy products [44] and saturated fats [36, 45]. On the flip side, foods such as fish [46], soy [47], cruciferous vegetables [48] and tea and coffee [49, 50] have been linked to a decreased risk of prostate cancer.

#### 2.4.5 Vasectomy

There have been numerous studies over the years that have investigated vasectomies and their possible link to cancer development. A study from Denmark showed that men who had a vasectomy were 15% more likely to develop the cancer than those who were not vasectomised [51]. Xu et al. [52] analysed 58 separate studies and compiled and assessed them to try and identify whether there was a connection between vasectomies and increased cancer risk. The 58 studies came from countries all around the world, and the results from this analysis demonstrated that there are potentially 6%–12% increased chances of all stages of cancer in vasectomised men compared to non‐vasectomized men, but no associated increased risk of mortality. These studies confirm that vasectomies are heavily linked to an increased risk of the development of prostate cancer but not so much to the risk of developing a more lethal variant of the disease.

#### 2.4.6 Genetic Factors Associated With Prostate Cancer

Genetic factors often associated with prostate cancer can be either hereditary or due to mutations such as indels, missense mutations, substitutions or frameshift mutations. So far, there have been several genes linked with hereditary prostate cancer, which include mismatch repair genes such as post‐meiotic segregation increased 2 (*PMS2*), MutS homolog 2 (*MSH2*) and MutL protein homolog 1 (*MLH1*), as well as recombination genes such as partner and localiser of BRCA2 (*PALB2*), Ataxia‐telangiectasia mutated (*ATM*) and Breast Cancer genes 1 and 2 (*BRCA1/2*) [53]. However, the commonality of hereditary mutations within mismatch repair genes was found to be significantly lower than those found in genes of other DNA repair pathways [54]. That being said, those individuals with these germline mutations are at a 2%–4% higher risk for prostate cancer development than those without these mutations [55].

It may be strange to see the genes *BRCA1* and *BRCA2* here, as these are the model genes for hereditary ovarian and breast cancer. However, recent studies show increased risk in men with germline *BRCA* mutations, with men above the age of 65 with these mutations being 4–8 times more likely to develop prostate cancer than those without these mutations [56, 57]. *PALB2* is a gene known to be a link between the *BRCA1* and *BRCA2* genes, forming the *BRCA* complex [58]. Although uncommon, *PALB2* germline mutations are connected with a 0.6% increased chance of disease development [59, 60].

The *ATM* gene, found on chromosome 11, functions as a DNA damage repair [61]. It is well known that for an individual who has homozygous loss‐of‐function mutations for the *ATM* gene, the resulting outcome would be the disease known as ataxia telangiectasia syndrome [53]. However, a heterozygous carrier of this mutation was proven to have an increased likelihood of prostate cancer of around 6% [62, 63].

Moving on from hereditary prostate cancer, there are also those genes where somatic mutations can lead to the development of the disease. Mutations in *NKX3.1*, *Myc* and *PTEN* are some of the prevailing causes of prostate cancers [8, 64, 65, 66]. The homeobox gene NKX3.1 encodes a prostate tumour‐suppressing protein, making it an excellent marker for prostate cancer development [64]. If the expression of this gene were lost, it could result in errors in prostatic protein secretion and duct morphogenesis and ultimately lead to prostate carcinogenesis. This loss of expression can often result from promoter methylation, allele deletions or post‐transcriptional silencing [67, 68]. Biomarker development focusing on the expression of this gene could potentially be an excellent indicator for this form of cancer in its early phases.

The *Myc* gene family is made up of three genes, *n‐Myc*, *l‐Myc* and *c‐Myc* [69]. *c‐Myc* encodes a regulatory protein which, when overexpressed, can lead to the initiation and progression of various tumour types [70]. This overexpression of the *c‐Myc* gene is in association with alterations in the gene, such as amplification and translocations [70]. For this reason, the *c‐Myc* gene expression can make for an excellent biomarker in the progression and treatment of prostate cancer.

Genomic deletions can result in the *PTEN* gene losing its functionality. This gene encodes the phosphatase and tensin homologue (PTEN) [71]. When this tumour suppressor protein is lost, alterations are made to the PI3K‐AKT‐mTOR signalling pathway, which is often found to be associated with advanced prostate cancer stages with poor outcomes [72, 73]. There is the potential for personalised treatment plans involving the PI3K‐AKT‐mTOR pathway being inhibited, which shows potential for cancer treatment. Therefore, effective *PTEN* biomarkers may be a valuable asset in the future [73].

### 2.5 Progression of Prostate Cancer Development

**FIGURE 6:**
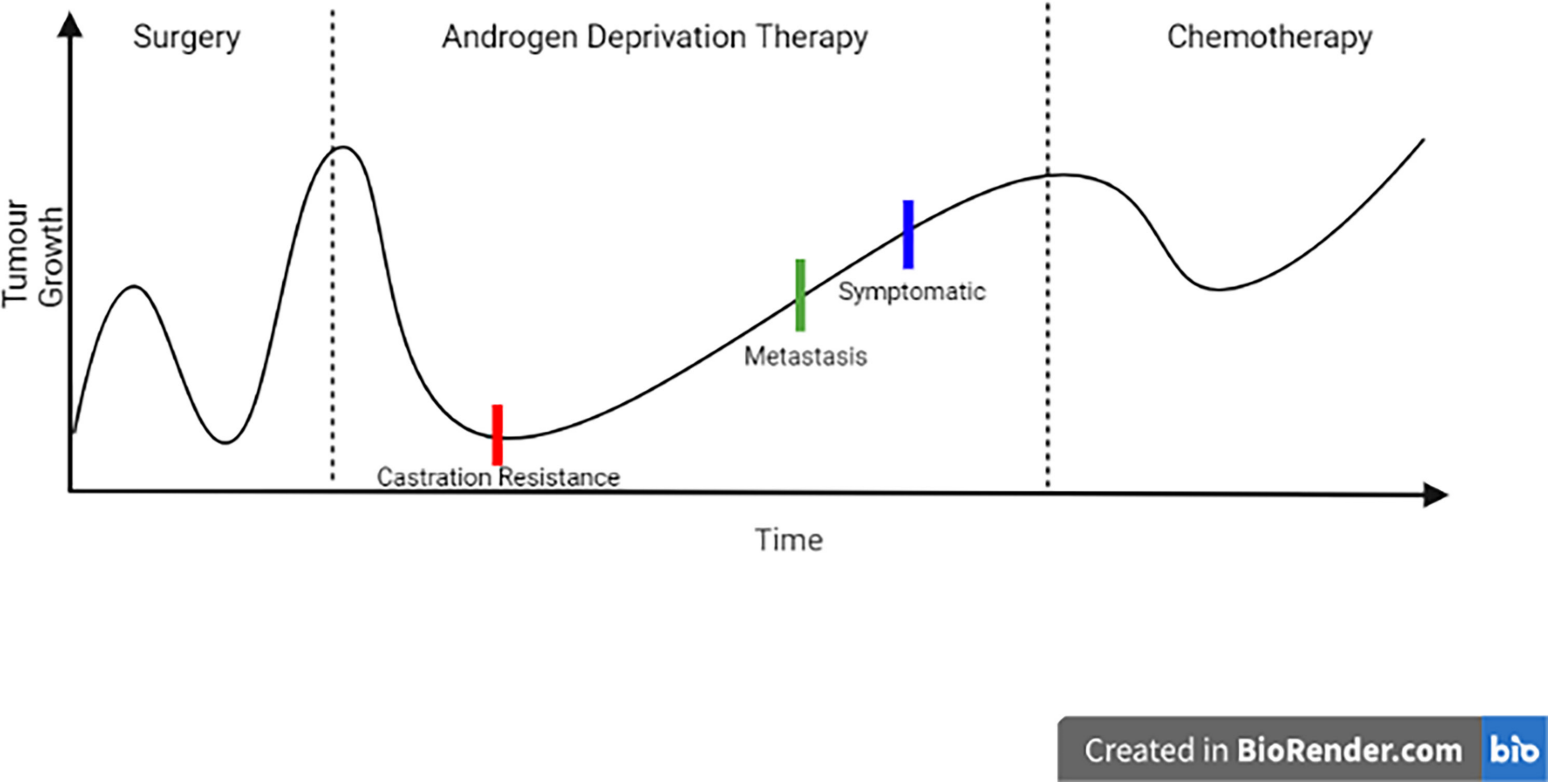
Progression of prostate cancer over time, showing the different general therapies used and at which stages the cancer becomes castration‐resistant, metastatic and symptomatic. Created using www.BioRender.com (2023).

There is no exact mechanism for prostate cancer development, but there is a generalised flow of events, which is represented in the figure below (Figure 6). For the most part, prostate cancers are identified before they spread to other bodily organs, allowing for localised therapies to be put in place, such as surgical removal of the tumour mass and radiotherapy [74]. If the disease continues or relapses after initial treatment, the next step would be the use of Androgen‐deprivation therapy (ADT). However, even though this therapy may be effective at first, almost all patients eventually stop responding after a few years, leading to the cancer becoming castration‐resistant [75, 76]. From this stage, the cancer progresses and spreads, becoming metastatic and symptomatic, leading to the need for chemotherapy treatment to be put in place [77]. Unfortunately, if this final treatment method does not work, the eventual outcome for the patient is death.

## 3 Diagnostic and Screening Methods for Prostate Cancer

**FIGURE 7:**
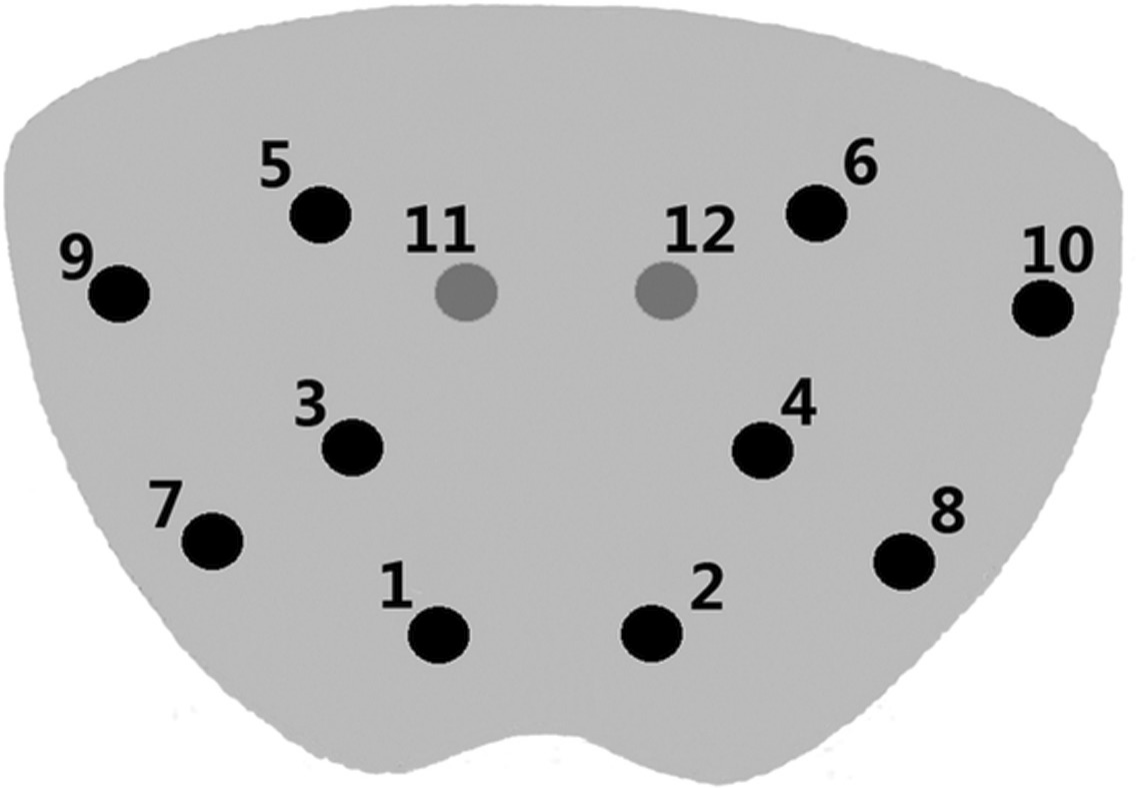
Transverse section of the prostate indicating the positions from which the 12 cores are taken: 1–6 are the traditional sextant, 7–10 come from the peripheral zone and 11 and 12 come from the transition zone. *Image Source:* [81] (Open License CC BY).

Traditionally, prostate cancer is diagnostically confirmed through biopsy and evaluation of prostate tissue at the microscopic level, known as a 12‐core systematic biopsy [78] (Figure 7). The biopsy is performed as follows: 12 tissue samples are collected with the aid of transrectal ultrasound and examined, and a Gleason primary and secondary grade between 1 and 5 is issued for the most common and highest histological pattern. This grade is given by judging the appearance of the cells. Looking at both scores together gives a combined score out of 10, which is used to determine the risk level of the disease. Gleason scores less than/equal to six indicate either a very low risk or a low risk, depending on if the tumour is at stage T1c or T1‐T2b. Intermediate risk is defined as a Gleason score of 7 with tumour stage T2b‐T2c. High risk and very high risk share the same Gleason score of 8–10 but differ in tumour stage, with high risk having a T3a stage and very high risk having a T3b‐T4 stage [79]. Even though this systematic biopsy has proven the test of time and has been the most commonly used diagnostic method for many years, it has been known to have inaccuracies when classifying the cancer as well as missing cancer diagnoses altogether [80]. Because of this, new diagnostic methods have been studied.

### 3.1 MRI‐Targeted Biopsy

One such method explored is the MRI‐targeted biopsy. The issue found in the systematic biopsy is that it samples the prostate tissue systematically, with no targeting methods in place to target specific cancerous tissue locations in the patient, which may be seen as a shortcoming of this biopsy method [80, 82]. MRI‐targeted biopsies help to alleviate this problem using magnetic resonance imaging (MRI). Initially, the patient has their prostate imaged through this technique, and the images are interpreted by a radiologist who will identify lesions to be targeted for sample collection during the biopsy [83]. Ahdoot et al. [80] conducted a study in which both MRI‐targeted and systematic biopsies were performed in conjunction. The results indicated that MRI‐targeted biopsies revealed significantly lower diagnoses of grade 1 cancers but significantly higher diagnoses in grades 3–5 cancers. Combined biopsies led to fewer upgraded cancer statuses in men who underwent radical prostatectomy than either biopsy on their own. These results show that both methods have disadvantages but can result in more accurate cancer diagnoses when used together.

### 3.2 Liquid Biopsy

The two previous biopsies mentioned required particularly invasive techniques for sample collection, which many men may not agree to despite the risks imposed if not undertaken. Because of this, the development of less invasive diagnostic procedures has been investigated. One such method is a liquid biopsy targeting extracellular vesicles released by tumour cells [84]. Liquid biopsies solve the problem of invasiveness of the previous two diagnostic approaches and can also be used to monitor changes in the tumour in real time thanks to the circulation of the biomarkers within the body fluid [84]. Besides using extracellular vesicles, other biomarkers such as cell‐free DNA or free tumour cells can be used for liquid biopsies in diagnosing prostate cancer [85, 86]. However, it has been shown that extracellular vesicles are more significant in cancer diagnosis when compared to other biomarkers used in liquid biopsies [87].

## 4 Prostate Cancer Genes

The genetics of prostate cancer were previously alluded to in this review. The following section will address a more in‐depth look at the different genes linked to the onset and progression of this disease. There have been associations made between prostate cancer and many different types of genes, including those previously articulated, as well as genes from the families like erythroblast transformation specific (ETS) and *Myc* family, along with genes from the androgen receptor (AR) signalling pathway, and genes involved in pathways for DNA repair [7]. This section, therefore, aims to unpack more information on these genes.

### 4.1 Gene Families

The Erythroblast Transformation Specific (ETS) and MYC families are prominent gene families associated with prostate cancer development. The ETS family comprises transcription factors that arise from the encoding of 28 genes [88]. The defining feature of all ETS transcription factors is conserved ETS DNA binding domains organised in a winged helix‐turn‐helix [89].

ETS family members can be found throughout the body and perform multiple functions, including lymphoid cell development, cell differentiation, and/or proliferation, along with cell death (apoptosis) [90]. However, some ETS factors function as oncogenes and tumour suppressors. Members of the PEA3 (*ETV5*, *ETV4* and *ETV1*) and ERG (*FEV*, *FLI1* and *ERG*) sub‐families can behave as oncogenes [89], with evidence of oncogenic expression in ERG and PEA3 genes occurring early on in prostate cancer development. However, it is not involved in the initiation of the disease itself but rather in conjunction with other factors, such as *PTEN* loss [91, 92].

The MYC gene family has three major members, including *L‐Myc, N‐Myc* and *c‐Myc* [93]. These three genes are connected with multiple processes at the cellular level, including differentiation, metabolism and cellular division and death [94]. From this family, the one associated most often with the disease in question is *c‐Myc*. *c‐Myc* is found on chromosome 8q24, and in healthy prostate cells, its expression would be regulated by mitogenic signals [93]. However, the problem arises in cancerous cells, where the gene becomes overexpressed. This overexpression was identified in the early stages of prostate cancer development [95, 96]. In its regular state, however, *c‐Myc* encodes a transcription factor connected with cellular function regulation, including growth, apoptosis and proliferation [97].

### 4.2 Tumour Suppressor Genes

As their name states, the normal function of tumour suppressor genes is to prevent cell proliferation, leading to tumour formation [98]. There are several tumour suppressor genes regularly involved in prostate cancer development, which include *pten*, *RB1* and *TP53*, with alterations in these genes leading to cell cycle aberrations [99, 100]. Analysing metastatic castration‐resistant prostate cancer has revealed a close association between resistant prostate cancers and these tumour suppressor genes, indicating that the role these genes play in resistance is large [101].

*TP53* encodes the tumour protein p53. Initial studies of this gene's association with prostate cancer showed its inactivation to be highest in the late stages of the disease, with metastatic castration‐resistant prostate cancer being heavily linked to these mutations [102, 103]. However, since then, new evidence has suggested this gene's mutations are present in greater frequency than initially believed within the early stages of cancer, along with metastatic castration‐naïve cancer [101, 104, 105]. *RB*1 encodes tumour‐suppressing pRB proteins [106], the inactivation of which is often found in Neuroendocrine prostate cancer (NEPC), an aggressive subtype of the disease [107]. *RB1* inactivation is often found in conjunction with *TP53* mutation to result in the driving force behind NEPC [108, 109]. Although both gene's mutations are present in both NEPC and castration‐resistant prostate cancer, *RB1* mutations can be found in much higher frequencies in NEPC than in the castration‐resistant form of the disease [110].

**FIGURE 8:**
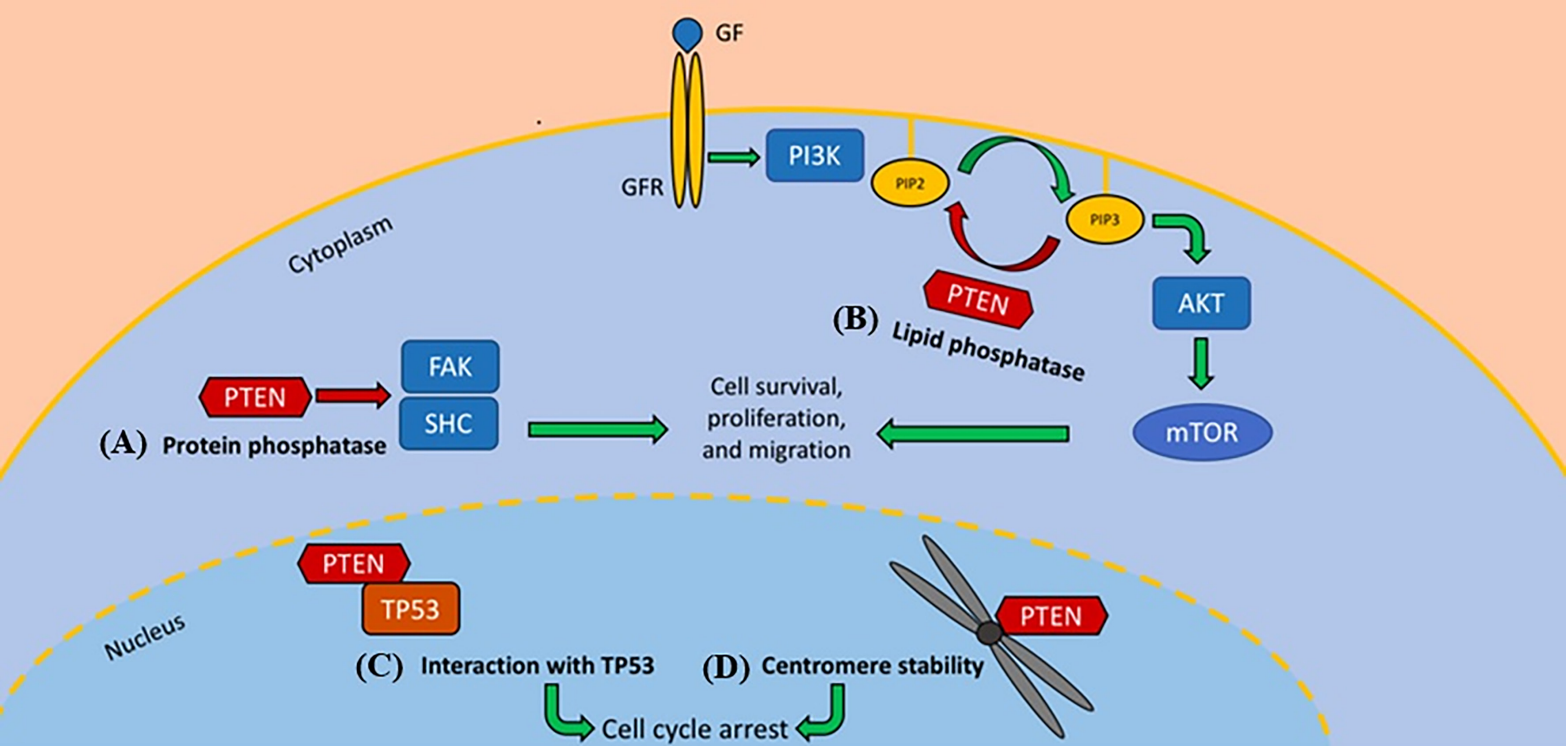
Diagrammatic representation of the functions of the PTEN tumour suppressor, showing the role it plays in regulating the conversion of PIP_3_ to PIP_2_ and preventing the phosphorylation of AKT, thereby preventing irregular mTOR activation and dysregulating cellular functions. (A) PTEN regulates cell migration through protein substrate dephosphorylation; (B) PTEN inhibits the PI3K/Akt signalling cascade through PIP3 dephosphorylation to PIP2; (C) PTEN interacts with TP53, resulting in cell cycle arrest through increased TP53 stability; (D) PTEN preserve chromosome stability through interaction with the centromere. *Image Source:* [113] (Open License CC BY).

A third tumour suppressor gene linked to this cancer is the phosphatase and tensin gene *pten*. With regards to prostate cancer development, this gene is among the most associated genes [71]. *pten*'s Loss‐of‐function mutations often lead to alterations within phosphoinositide 3‐Kinase (PI3K) pathways [71]. In healthy cells, the PTEN tumour suppressor protein functions in metabolising the PI3K lipid PIP_3_ into PIP_2_ [111]. In doing so, PTEN antagonises the class I PI3K enzymes, which serve to convert PIP_2_ to PIP_3_, leading to RAC‐α serine/threonine‐protein kinase (AKT) activation and rapamycin (mTOR) signalling cascades being mechanistically targeted [111]. If a loss‐of‐function mutation were to be present in the *pten* gene, there would be no PTEN present to convert PIP_3_ into PIP_2_, which would cause phosphorylated AKT to modify downstream targets, such as the mTOR signalling cascade, resulting in uncontrolled cellular function regulations, including cellular differentiation, proliferation, growth and apoptosis [112]. Figure 8 below shows the function of PTEN diagrammatically.

### 4.3 Androgen Receptor (AR) Signalling Genes

**FIGURE 9:**
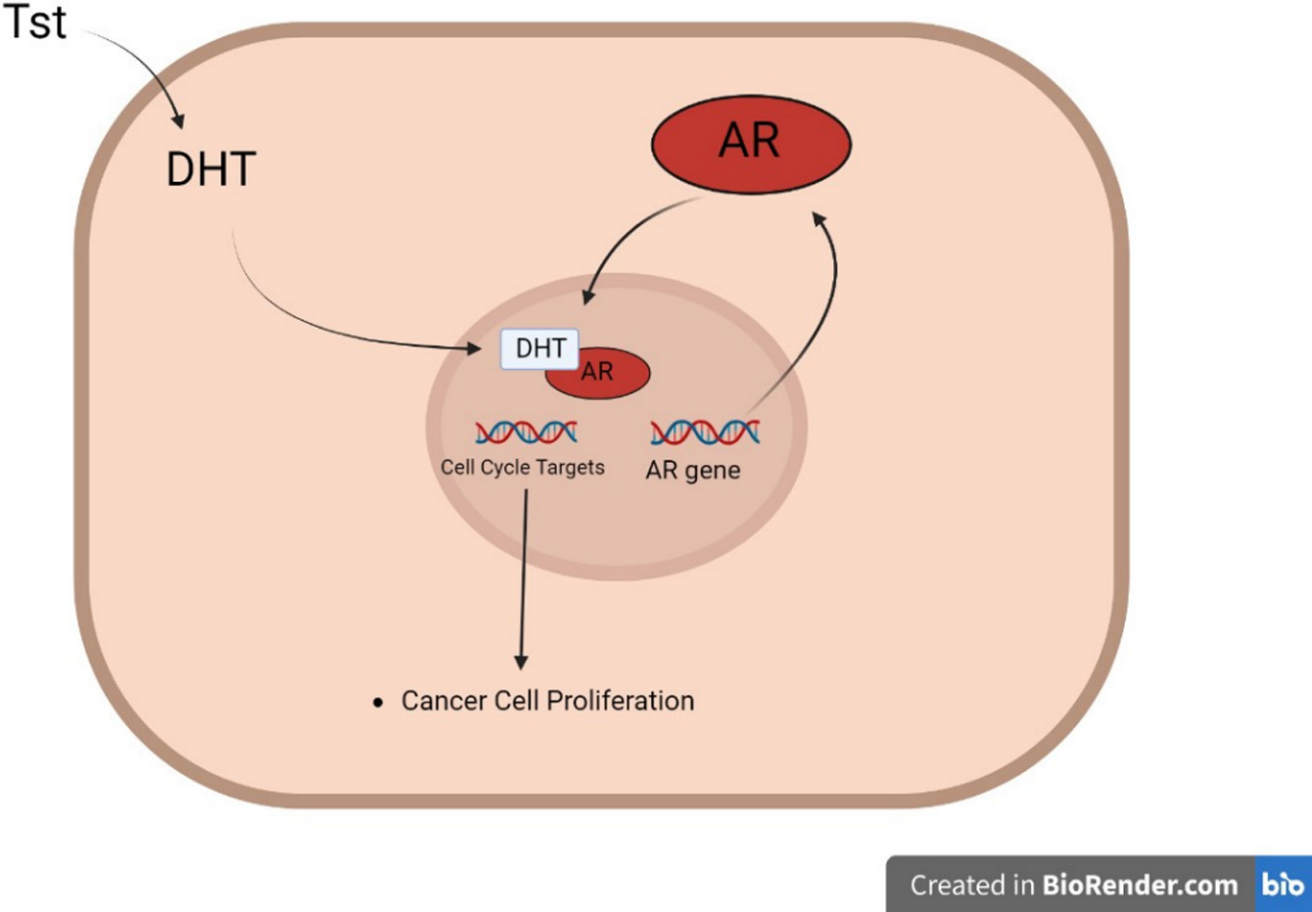
Process of prostate cancer cell proliferation through the dependence on testosterone (Tst) and its conversion to dihydrotestosterone (DHT), which binds with the AR, where it then moves to the nucleus, causing cell cycle targets transcription, resulting in cancer cell proliferation. Image created using BioRender (www.biorender.com). Inspiration from [118].

The AR is found in several tissues, and when unbound to a ligand (androgens), it remains within the cytosol, attached to heat shock proteins [114]. In males, the binding of androgens to ARs instigates the individual's sexual development. In contrast, it maintains several mechanisms for adult males, such as spermatogenesis, libido, bone mineral density and muscle mass [114, 115]. Healthy prostate epithelial and stromal compartments express AR [116]. In cancerous prostate cells, the regular AR signalling is disrupted, and proliferative AR signalling occurs, with the exact mechanism responsible for this switch being unknown [114, 116]. However, studies show that cancer growth in the prostate depends on testosterone, which converts to dihydrotestosterone (DHT). This conversion occurs when testosterone is reduced by the enzyme 5‐alpha‐reductase, resulting in a double bond being removed from the testosterone molecule, forming DHT [117]. DHT binds with an AR, leading to the translocation of this DHT‐AR complex into the nucleus and increases the cell cycle genes' transcription rates, resulting in the proliferation of cancer cells [118] (Figure 9).

Because of this cancer's strong association with AR signalling, androgen deprivation therapies (ADTs) were developed to help curb the growth and development of the disease. However, resistance to these therapies is inevitable and causes severe issues regarding treatment [114]. Three major mechanisms lead to ADT resistance, the most common being reactivation of the AR signalling pathway despite deficient levels of androgens being present, which can occur through AR protein over‐expression or AR gene amplification [119]. The other two mechanisms of ADT resistance include the bypass mechanism, which allows AR‐dependant pathways to activate regardless of AR‐activation, and the de‐differentiation of prostate cancer cells into NEPC cells [120, 121]. This resistance to ADTs is what leads to regular forms of cancer progressing to the castration‐resistant form of the disease.

### 4.4 DNA Repair Genes

**FIGURE 10:**
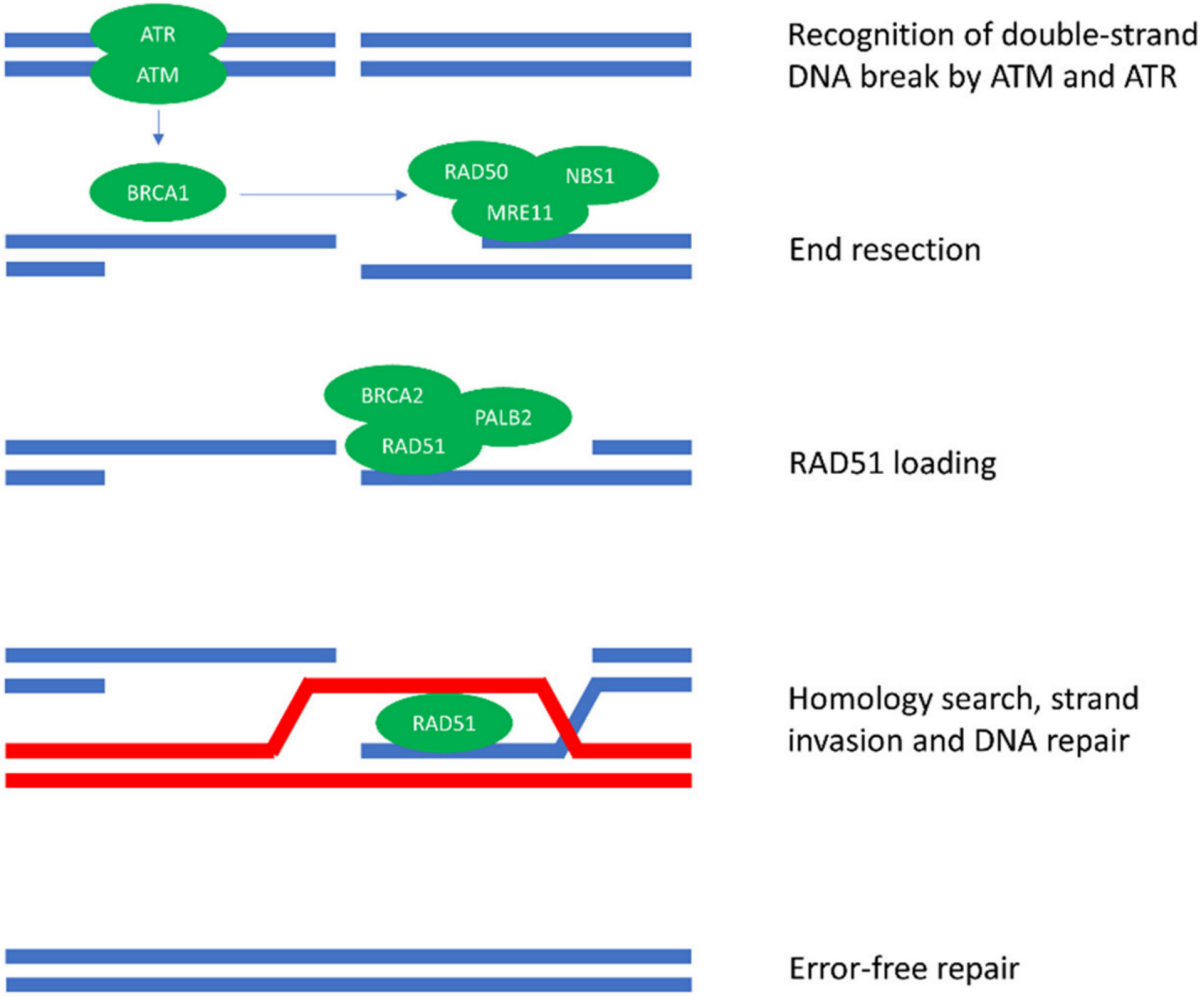
Diagram showing the process of DNA repair through homologous recombination controlled by multiple DNA repair proteins. *Image Source:* [124] (Open License CC BY).

The BRCA2 protein drives homologous recombination, an extremely accurate method of DNA repair that uses the sister chromatid to template new DNA synthesis, reducing the risk of DNA aberration accumulation [122, 123]. However, loss‐of‐function or deleterious mutations can cause this highly accurate homologous recombination to be lost or impaired, resulting in less precise DNA repair mechanisms being relied upon, leading to possible tumorigenesis [122]. However, BRCA2 is not the only protein involved in DNA repair through homologous recombination. The process begins with the ATM and ATR proteins recognising damaged DNA segments, activating BRCA1, which recruits proteins for end resection. RAD51, assisted by PALB2 and BRCA2, is loaded onto the end strand of DNA. This end strand invades the sister chromatid's homologous DNA strand to ensure that the repair of the DNA can now take place [124] (Figure 10). The removal of BRCA2 from this process will result in the aforementioned impairment of the homologous recombination process. If markers identifying these BRCA2 mutations were developed, treatment could be tailored for this, allowing for more effective, personalised treatment plans.

The *CDK12* gene encodes the cyclin‐dependant kinase (CDK) 12, a 1490 amino acid protein that, with cyclin K, creates a complex to regulate DNA damage repair proteins [125]. Cells with mutant forms of the *CDK12* gene can have a less functional homologous recombination activity than healthy cells [126]. However, studies have shown that cancers with CDK12 downregulation and inactivation are more susceptible to treatment through chemotherapeutic agents like cisplatin, camptothecin, mitomycin C or etoposide [125, 127].

**FIGURE 11:**
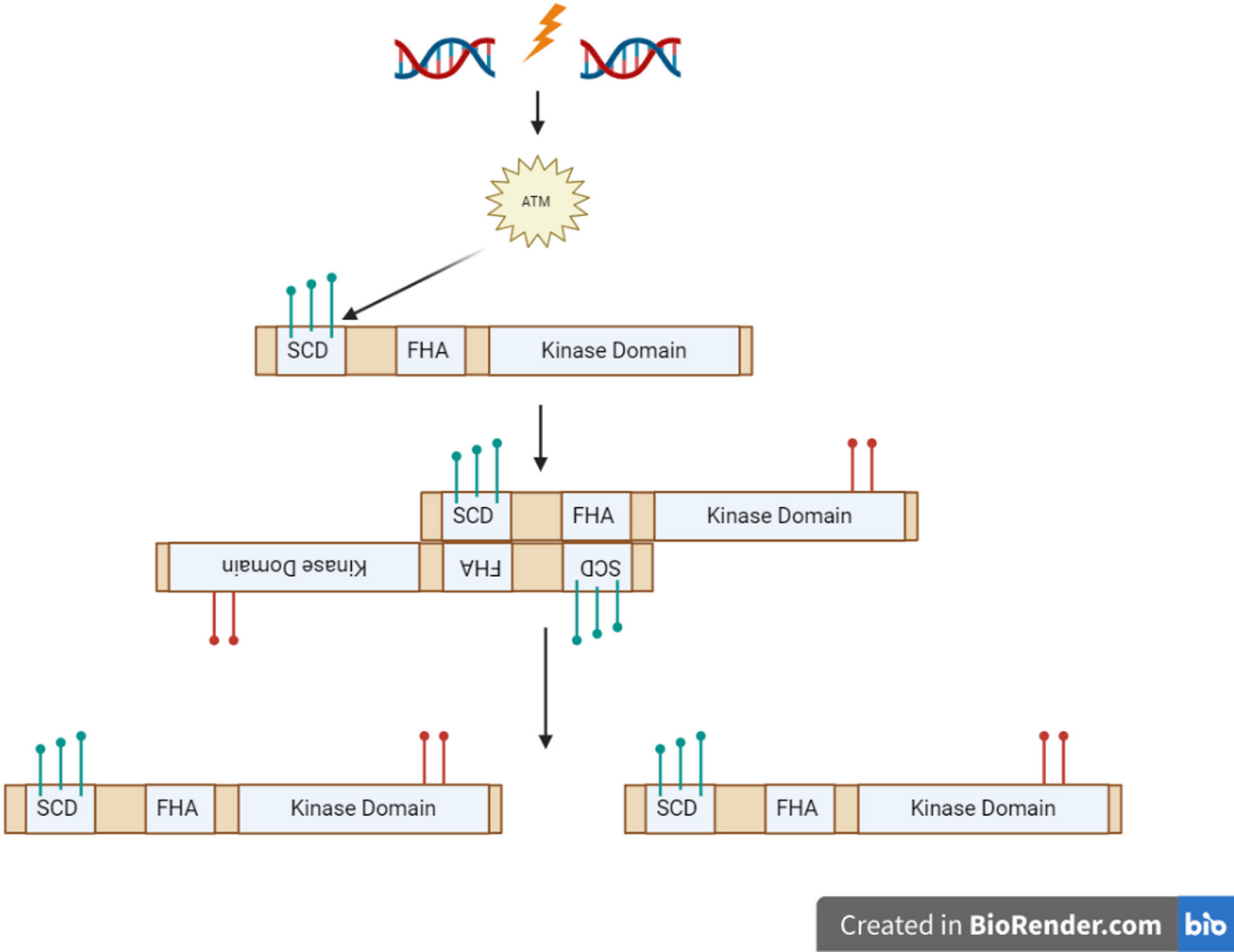
Activation Process of the CHK2 protein. Firstly, as DNA damage is identified, ATM triggers the phosphorylation of the SQ/TQ cluster domain (SCD) of CHK2, leading to dimerisation and autophosphorylation of the kinase domain, triggering conformational changes resulting in dissociation and activation of monomer CHK2 proteins. Image created using BioRender (www.biorender.com).

The *CHEK2* gene encodes the checkpoint kinase 2 (CHK2), which is essential to the ATM‐CHK2‐P53 pathways [128, 129]. When DNA is damaged, CHK2 protein phosphorylated at the priming site and SQ/TQ cluster domain (SCD), by ATM, takes place [130]. This phosphorylation leads to the dimerisation of CHK2, which in turn phosphorylates the kinase domain of the CHK2, which again leads to conformational changes resulting in the dimers dissociating into active monomers [130] (Figure 11).

The active CHK2 protein can be found to be associated with the repair of DNA through phosphorylating BRCA1 and 2, causing homologous recombination of double‐stranded DNA breaks [130, 131]. Another method through which the CHK2 protein is involved in DNA damage repair is through a mechanism known as base excision repair. Through this mechanism, damaged DNA nucleobases are recovered, starting with the activation of the FoxM1 (Forkhead Box protein M1) by a phosphorylated CHK2 protein, which leads to XRCC1, a base excision repair factor, being transcribed [132].

Taet al. [133] demonstrated that CHK2 activity loss is adversely related to Androgen Receptor activity, resulting in increased cell proliferation. What this shows is the role played by CHK2 in Androgen Receptor regulation, linking DNA damage repair with prostate cancer growth and proliferation through Androgen Receptor mediation. Therefore, finding treatments that could regulate CHK2 activity can result in downstream AR regulation and more effective care being available.

## 5 Genome‐Wide Association Studies Into Prostate Cancer

Over the years, there have been several GWAS studies performed focusing on finding a link to prostate cancer and how to treat the disease best. Through these studies, over 170 common genetic variants have been identified to be linked with prostate cancer [134]. Many of these single nucleotide polymorphisms (SNPs) identified lie within non‐coding regions of the genome, while others are found within coding regions [134]. An example of some SNPs found in non‐coding DNA regions comes from those identified in the 8q24 region. This region does not encode any proteins, and its nearest gene is the *MYC* gene [134]. It is believed that genetic aberrations at this location can result in influenced expression within the *MYC* gene, resulting in prostate cancer development [134].

A study by Du et al. [135] revealed that the 8q24 locus can have inter‐chromosomal interactions, with one of these interactions involving the *CD96* gene on chromosome 3q13. *CD96*, which encodes a membrane protein involved in immune response through interaction with T and NK cells, is believed to have an association with prostate cancer development through interactions with pathways involving mIR‐127, a miRNA associated with cancer [136].

There have been GWASs looking to find associations in mutations in men of different ethnicities and prostate cancer. Hoffmann et al. [137] conducted a multiethnic GWAS, where they identified new risk variants and determined the relationship between risk variants and ethnicity. This study identified a new risk indel, rs4646284, at location 6q25.3. This study also revealed that from the study group, men of African ethnicity had the highest frequency of this variant, along with the highest odds ratio (OR) associated with this variant, meaning that they have a higher chance of disease when this variant is present than any other ethnicity [138]. African men had a frequency of 0.365 and an OR of 1.18. The next highest OR was found in Caucasian men, with an OR of 1.17 but a frequency of only 0.298. The lowest OR was found in Asian men, with an OR of only 1.03, but the second highest frequency of 0.324, showing that this risk variant is less likely to cause disease in Asian men than men of other ethnicities. This is in line with the expectation that Asian men are less likely to develop prostate cancer than men of other ethnicities.

## 6 Current Biomarkers Associated With Prostate Cancer

Biomarkers or ‘biological markers’ is a very broad term associated with different signs that may be utilised and measured with both accuracy and repetition amongst all patients [139]. These signs differ from symptoms, described as indications of illness that the patient can see and feel. Biomarkers, in general, can range from simple tests and indicators like blood pressure and heart rate and simple tests such as cholesterol and blood sugar levels to more advanced laboratory tests involving many sample origins, such as blood, tissue, urine and others. Biomarkers aid both the development as well as the choice of personalised treatment regimens for individual patients along with assisting in reducing the amount of unnecessary medical procedures, many of which are invasive and uncomfortable to the patient, performed daily, providing patients with the ease of mind that they are receiving the best medical advice they can get.

The most widely used and accepted prostate cancer biomarker currently available commercially is the prostate‐specific antigen (PSA) test, which is recognised to be the predictor of choice for prostate cancer diagnosis and screening for many years [140]. This being said, that does not mean that this marker does not have its disadvantages. Studies as far back as 2003 have shown large numbers of false positives coming from the PSA test, with Mistry and Cable [141] showing that PSA tests can have as low as a 25% positive predictive value, resulting in 75% of patients undergoing the extremely invasive prostate biopsy previously eluded to.

For this reason, alternate techniques and biomarkers have been researched to try and find improved methods for diagnosing and screening prostate cancer. Several different biomarkers have been identified as possible alternatives or additives to the PSA test over the years, including serum biomarkers, urinary biomarkers, tissue biomarkers and genetic biomarkers [140, 142, 143]. A few biomarkers from each subtype will be discussed further here.

### 6.1 Biochemical Markers

Biochemical markers (serum biomarkers) refer to those markers whose quantitative values change within the blood serum during tumour development and growth [144]. The PSA test is a serum biomarker, as the PSA is a protein found within the blood serum. However, other serum biomarkers have been researched, including the prostate health index (PHI), which develops from the original PSA test. The PHI uses a formula that combines all three major PSA isoforms, namely p2PSA, free PSA and total PSA, into one score which has utilisation for predicting prostate cancer [140, 145]. Recent studies have shown that the use of a PHI test could reduce unnecessary prostate biopsies by up to 40% and assist in prostate cancer progression assessment during active surveillance [146, 147].

Another biochemical marker is the 4 K score, which utilises kallikrein‐related peptidase 2 (hK2), free PSA, total PSA and inactive PSA [140]. Similar to the PHI test in that it uses different PSA‐based markers, the 4 K score differs by also introducing other patient characteristics such as age, results of a digital rectal examination and any prior biopsy history, and combining all factors to create an algorithm to determine if there is any risk of prostate cancer of clinical significance [140]. Zappala, Scardino [148] showed that the 4 K score test was excellent at distinguishing clinically significant prostate cancer in pre‐biopsy settings, allowing for a drastic reduction of unnecessary biopsies being conducted.

### 6.2 Urine‐Based Biomarkers

Several biomarkers based on urine samples have been developed recently, including a urine exosome test, ExoDx Prostate IntelliScore (EPI), developed by Exosome Diagnostics Inc. (Waltham, MA, USA). What this test does is read the levels of exosomal expression of the *PCA3*, *SPDEF* and *ERG* genes' RNAs to predict the possibility of higher‐grade prostate cancers in patients [149]. Exosomes are extracellular vesicles that encapsulate parental cell cytoplasm containing high levels of cellular RNAs and proteins, which can be highly representative of parent cell profiles [150, 151]. McKiernan et al. [149] showed that using the EPI test conjointly with other factors like PSA, age and familial history led to a more accurate diagnosis than using only one variable. This test could reduce unnecessary prostate biopsy usage in the future.

The next urine‐based biomarker is the SelectMDx test (MDx Health, Irvine, Irvine, CA, USA). The SelectMDx test utilises urine samples collected after the digital rectal examination (DRE) has been performed to measure mRNA levels of the *HOX6* and *DLX1* genes [140]. These two genes are involved in prostate cancer onset and aggressiveness [152]. Van Neste et al. [153] showed that the use of the SelectMDx test reduced unnecessary prostate biopsies by up to 50%, demonstrating its value in a real‐world scenario.

The Progensa PCA3 (Gen‐Probe Inc., San Diego, CA, USA) test is another type of urinary biomarker for prostate cancer. The Progensa PCA3 test utilises post‐DRE urine samples to detect the prostate cancer gene 3 (PCA3). It is believed that this gene can be expressed up to 80 times more in prostate cancer patients than in non‐cancer patients [143]. This test determines the PCA3 mRNA to PSA mRNA ratio [154]. If this test returns a result under 20, this indicates a low cancer risk, whereas a result above 35 indicates a higher cancer risk and a biopsy should follow. Results between 20 and 35, the recommended course of action is a retest 6 months from the initial test [155].

### 6.3 Tissue‐Based Biomarkers

Along with the SelectMDx test, MDX Health has a tissue‐based biomarker known as the ConfirmMDx test. The ConfirmMdx test takes place after the initial prostate biopsy in which the results came back negative for prostate cancer, but PSA levels are still high [140]. In essence, the initial cancer‐negative tissue samples collected from the initial biopsy are used in this test, where DNA is extracted and used in a multiplex PCR assay aimed at measuring the epigenetic status of the *RASSF1*, *APC* and *GSTP1* genes, all of which play roles in cellular function and are linked to development of this disease [156, 157, 158].

Many tissue‐based biomarkers for prostate are used more for prognosis rather than diagnosis, as these biomarkers require tissue samples often collected during prostate biopsy or radical prostatectomy. One such prognostic biomarker is the Promark test developed by Metamark (Cambridge, MA, USA). This test is recommended for patients whose previous biopsy results achieve a Gleason score of 3 + 3 and 3 + 4. It uses the tissue sample to estimate the protein signatures of eight different proteins and predicts how aggressive the patient's cancer will be [143]. Predictions can be used to determine whether aggressive treatment is necessary.

Several tissue samples can hold untapped potential for possible biomarkers. One such form is that of microRNAs (miRNAs) [159]. These miRNAs are short (21–25 nucleotides) stretches of non‐coding RNAs which are believed to play a role in gene regulation and have great potential to be used for cancer diagnosis and prognosis [160, 161, 162]. Several miRNAs have already been identified, but the full extent of their relationship with prostate cancer development remains unknown. These include the MiR‐30 family, MiR‐451 and MiR‐218‐5p, to name a few [159]. If these miRNAs were to be studied more in‐depth, they could become valuable assets as biomarkers for prostate cancer, revealing a necessity for further research.

Protein S is typically involved in anticoagulation [163]. However, studies have shown that late‐stage prostate cancer has elevated expression of this protein, as it shows signs of regulating cell migration and proliferation in tumour cells [164]. This protein can provide valuable insight for personalised treatment regimens, as high levels of protein S have been found to cause resistance to treatment which stimulates prostate cancer cells through cAMP or IL‐6‐inducing agents [165]. Therefore, biomarker development focused on Protein S can assist in determining which treatment will be ineffective for an individual patient.

## 7 Biomarker Development: Techniques Used for Identification and Production of Novel Biomarkers

Although the biomarkers above seem plenty in the fight against prostate cancer, there is always still the need for the development of novel biomarkers which can be used in different stages of cancer progression, whether it be diagnosis, prognosis or the selection of treatment methods [166]. Biomarkers may be developed to determine whether or not a patient does indeed have prostate cancer, or they may be designed to assess the severity of the cancer currently present in the patient. A third use for biomarkers may be to determine the best course of treatment for individual patients, as certain patients may have mutations that make their form of cancer more resistant or susceptible to specific therapies, allowing physicians to choose the best treatments for particular patients.

The processes for biomarker development remain consistent throughout, with several steps followed to develop novel biomarkers. This process typically follows four main steps, those being the discovery of a possible novel biomarker, followed by assay development and analysis, clinical validation and finally its implantation in clinical practice [167].

### 7.1 Identification of Possible Biomarkers

Biomarker discovery is the first step in the development of biomarkers. However, this step is more complex than it may appear. Before one can go off and decide on where to aim to discover this novel biomarker, limitations and study settings must first be established [167]. Several factors must be considered before initialising the search for novel biomarkers, such as the target group for which the biomarker should target, the effect the biomarker should have (diagnostic, prognostic, etc.) and the sample type from which the biomarker will be tested. Often, bias comes into play when deciding these factors before research may begin, leading to the stunting of biomarker development [168].

To avoid this stunting of progress, the study design must be thoroughly discussed before deciding on the final methods. The study's region should be considered when deciding on the target group. Aspects such as ethnicity, age and sex are all important factors to consider before sample collection and analysis. However, it has been common practice for years that the samples used in biomarker discovery were samples that had already been collected for previous studies and were readily available for analysis [167, 168]. Samples used are often those previously analysed for different studies and taken for use in a new study focusing on biomarker discovery, resulting in less favourable conditions for biomarker discovery. Along with this issue of the reuse of samples, if samples are processed for biomarker discovery differently from how they were processed for the previous study, this could result in misleading information, as shown by Hernández, Parnell and Pennington [169].

### 7.2 Assay Development and Validation

A novel biomarker should be discovered if all study design aspects and methods were followed correctly. Following this discovery, an assay should be developed to repeat the identification of this marker for future clinical use if the marker and assay pass validation. The assay should be clinically suitable, meaning it can be used in healthcare settings [167]. The type of assay used will depend on the marker itself. If the marker should be a mutation or over or under‐expression of a gene, then typically the assay used would be a real‐time PCR (qPCR), whereas those markers that are either translocations or copy number‐related, then Fluorescent In Situ Hybridization (FISH) is ideal. Both of these types of assays will be discussed further below.

#### 7.2.1 Real‐Time Polymerase Chain Reaction

The original PCR has been around since 1984, when Karry Mullis developed the technique, allowing significant research breakthroughs. The PCR technique is now standard practice for several laboratory applications globally [170]. The premise of the PCR is, as the name states, a chain reaction resulting in the amplification of the target sequence. Essentially, three steps make up the PCR process, namely Denaturation, Annealing and Extension. During denaturation, the DNA sample is heated up, resulting in the bonds between the two strands of DNA breaking, causing the two strands to separate and become two separate single‐stranded DNA molecules. Following this is the annealing phase. During annealing, the RNA primers attach to the single‐stranded DNA at the annealing site they are designed to attach to. Once the annealing stage is complete, the extension stage begins. During extension, DNA polymerase attaches free DNA nucleotides to the single‐stranded DNA, forming two new double‐stranded DNA molecules from the original DNA strand. These three steps are repeated in cycles, with every cycle doubling the number of target sequences. Therefore, after 30 cycles, it is possible to have as many as 1 billion copies of the target sequence. This is the basic process of PCR, from which the real‐time PCR (qPCR) is built. After completion of the cycling process, the DNA is taken and viewed through gel electrophoresis.

Real‐time PCR, also known as quantitative PCR (qPCR), takes this original PCR process and negates the need for gel electrophoresis after the fact. With qPCR, a fluorescently labelled reporter dye is added to the reaction mix, which is used to measure the nucleic acid concentration during the PCR process quantitatively. This reporter dye is found at the 5′ end of a third internal primer known as a TaqMan probe [171]. On the 3′ prime end of the internal primer, there is a quencher dye, and when the quencher dye and the reporter dye are in close proximity, FRET (fluorescent resonance energy transfer) occurs, resulting in no observable fluorescence. However, during extension, as Taq DNA polymerase extends along the amplicon, it reaches the 5′ end of the internal primer and nicks the reporter dye free, causing no FRET to occur and fluorescence to be observed [171, 172].

#### 7.2.2 Fluorescence In Situ Hybridization (FISH)

FISH is another technique utilised to detect and locate specific DNA sequences within a chromosome. The technique runs as such: A DNA probe is designed where it will anneal to the target DNA sequence. Once the design is complete, the probe is labelled with a fluorescent dye. Following labelling, both the target sequence and DNA probe are denatured. Annealing and hybridisation of the probe and target sequence then occur, allowing researchers to view the location of the target sequence on the chromosome [173].

Over the years, FISH assays have been used extensively in biomarker research for many cancers and other human diseases [173]. Chromosomal rearrangements to genes in the TMPRSS2 and ETS families have been found in many cancer patients [174, 175]. Because of this, FISH assay development has occurred, with several assays being developed over the years targeting these specific chromosomal aberrations [174, 176, 177].

More common FISH assays used for prostate cancer target *PTEN*. *PTEN* loss‐of‐function occurs early on in this cancer development, occurring in up to 60% of localised prostate cancers [178]. *PTEN*, found on chromosome 10, is positioned between the *BMPR1* and *FAS* genes. Analysis of the loss‐of‐function can be performed using research conducted by Yoshimoto et al. [179], who, in their research, developed a four‐colour FISH assay. The four coloured probes used in this assay are as follows: a control probe which targets the centromere region of chromosome 10, which is labelled with a red dye; probes which target the *BMPR1* and *FAS* genes, labelled with green and blue dyes, respectively; and finally, a probe which targets the *PTEN* gene, labelled with a yellow dye. Including the two flanking genes in this assay helps to alleviate the possibility of false‐positive results. If all four colours are present when viewing the results, this indicates that there is no *PTEN* deletion or loss‐of‐function, but if the yellow signal does not appear but the other colours are all there, then *PTEN* deletion and loss‐of‐function has occurred, which could infer that prostate cancer has developed [178].

Before any assay is produced and used clinically, it must undergo analytical validation. This analytical validation refers to the reliability of the assay, along with its accuracy in measuring the desired target. For an assay to pass analytical validation and move on to clinical validation, it needs to be able to reproduce results consistently [167]. If the assay does this, it may proceed to clinical validation.

### 7.3 Clinical Validation of Assays

Clinical validation of the biomarker assay is done ideally through clinical trials, where the assay and its performance may be evaluated extensively [180]. However, this is only sometimes the case, as clinical trials are often expensive, or there is a limited number of potential patients from which samples may be obtained. Because of this, studies often use samples from a biorepository, which allows costs to be brought down significantly, along with increasing the number of samples available for validation testing [167]. Other aspects looked at during the clinical validation process include the cost‐effectiveness of the new biomarker assay (i.e., whether the assay's effectiveness outweighs the cost of development) and comparative analyses against similar biomarker assays [167].

### 7.4 Clinical Implementation

After the rigorous validation process, the biomarker assay is not quite ready for widespread rollout. Although the assay is validated and prepared for use in clinical care, there are a few steps still required before its rollout, those steps being regulatory approval, commercialisation, clinical practice guidelines approval and medical aid coverage [167]. Firstly, the biomarker assay must be approved by a regulatory board such as the South African Health Products Regulatory Authority (SAHPA) or the Food and Drug Administration (FDA). This approval can be achieved through testing within an approved diagnostic laboratory. Commercialisation involves finding a company willing to mass produce and market the new biomarker assay, allowing the assay to find its way into the mainstream healthcare market. Before mass production, however, the assay needs to be approved by medical aid schemes for coverage to those patients who may require its use. If the new assay gets approved and recommended in clinical practice guidelines, this would improve the chances of commercialisation from a medical company, ultimately resulting in its final clinical implementation.

## 8 Personalised and Experimental Treatment Methods for Prostate Cancer

Presently, the world is undergoing rapid developments in the fields of healthcare and personalised medical treatments. Implementing high‐throughput technologies has launched translational bioinformatics, a science involving retrieving, processing and storing data for improving human health, into the forefront of medical informatics [181]. These technologies have helped develop personalised treatment plans for individual patients to maximise their chances of recovery. As some forms of prostate cancer result from different genetic mutations, these forms of the disease may be resistant to certain drugs and treatments, making it necessary to identify which cancerous mutations each patient has to determine which treatments will work best [182]. Here, several treatment plans based on specific preliminary criteria will be explored. However, it is essential to note that implementing personalised medicines in developing countries faces problems. These problems involve the need for more necessary medical infrastructure to implement individually personalised medication, which leads to this strategy taking a backseat in these countries, causing further increases in mortality rates compared to developed countries [6, 183].

### 8.1 Metabolism‐Based Treatment Plans

To understand the treatments for altered metabolism in this disease, understanding the different substrates in normal prostate metabolism and how they are utilised in a healthy prostate is needed. Different substrates involved in metabolism include citrate, glucose, fatty acids and glutamine [184]. In most healthy cells, citrate oxidation occurs as part of Kreb's cycle. However, as citrate is key in semen production, the prostate produces and secretes citrate instead of oxidising it [185]. In healthy prostate epithelium cells, glucose gets taken up and undergoes glycolysis, then converted into pyruvate. Most of this pyruvate enters the mitochondria, where it enters Kreb's cycle to produce the citrate needed for semen. At the same time, the remainder gets further broken down into lactate, which is then removed from the cell.

#### 8.1.1 Citrate‐Targeted Treatment

**FIGURE 12:**
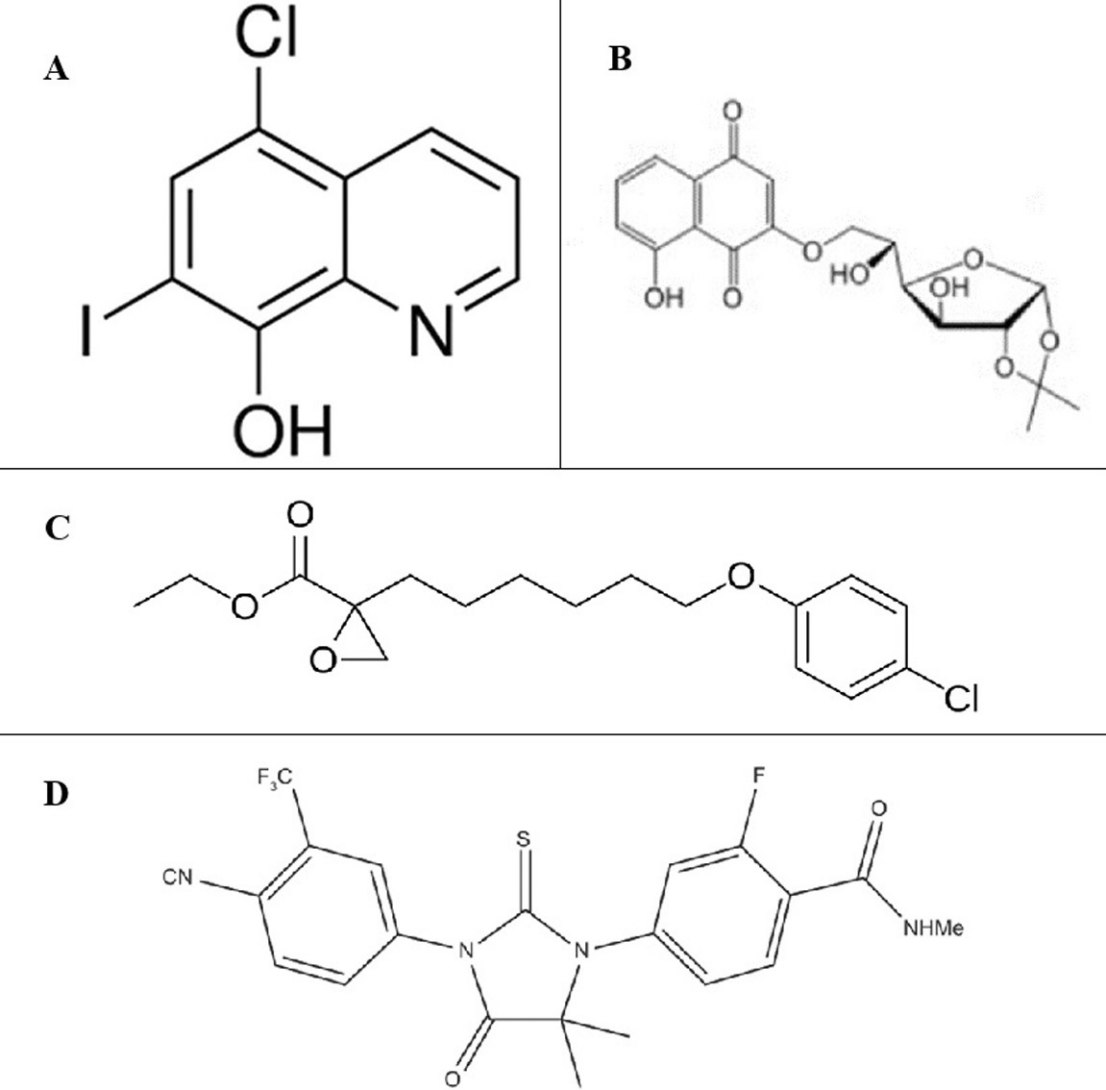
Chemical structure of several drugs used to treat prostate cancer. (A) clioquinol; (B) quinone‐glucose conjugate created through conjugation of a glucose molecule and 1,4‐naphthoquinone; (C) etomoxir; (D) Enzalutamide (4‐[3‐[4‐cyano‐3‐(trifluoromethyl)phenyl]‐5,5 dimethyl‐4‐oxo‐2‐sulfanylideneimidazolidin‐1‐yl]‐2‐fluoro‐N‐methylbenzamide). *Image Source:* [190, 191, 192, 193] (Open License CC BY & Public Domain).

A major prostate gland secretion is citrate, which forms part of the prostatic fluid [186]. In healthy prostate cells, ZIP1, a zinc transporter which the *SLC39A1* gene encodes, delivers zinc into the prostate. This process prevents mitochondrial aconitase activity, encoded by *ACO2*, which curbs citrate oxidation and Krebs cycle flux [182]. Due to this, the prostate gland's ATP synthesis relies on aerobic glycolysis and not oxidative phosphorylation, with the primary carbon donors being aspartate and glucose. However, in the case of cancerous prostate cells, ZIP1 expression is decreased along with citrate production, while ACO2 expression increases, leading to citrate‐oxidizing instead of citrate production in the prostate [187, 188]. This alteration results in increased energy production within the prostate, allowing for exponential proliferation and eventual metastasis. With this knowledge, theories of cancer treatment through a zinc‐based treatment plan could assist with inhibiting tumour growth [186]. The key to effective zinc treatment would be determining the ZIP1 activity in the cancer cell. Zinc treatment would only be effective if the cancer cells exhibited decreased ZIP1 activity. A possible drug potentially treating these malignant cells would be clioquinol (5‐chloro‐7‐iodo‐8‐hydroxyquinoline), a zinc ionophore (Figure 12). The clioquinol binds to plasma zinc when used, creating the zinc ionophore necessary for zinc delivery into ZIP1‐deficient cancer cells [186]. Franklin [189] conducted an animal study to determine the effectiveness of this treatment. The results showed an 85% decrease in tumour growth due to the effects of zinc in the cancerous prostate cells, showing a possible personalised treatment plan for those patients with a ZIP1‐deficient form of prostate cancer.

#### 8.1.2 Glucose‐Targeted Treatment

In all cells, glucose is a primary energy source, and its transport into cells leads to glycolysis and pyruvate production. Most of this pyruvate then enters the mitochondria, forming part of the Krebs cycle (TCA cycle or tricarboxylic acid cycle). During this cycle, the pyruvate undergoes oxidative phosphorylation, ultimately producing energy. In the absence of oxygen, glycolysis occurs in place of Kreb's cycle and oxidative phosphorylation, resulting in glucose ultimately being converted into lactate [194, 195]. As previously alluded to, normal prostate cells utilise glycolysis for energy production, but in cancerous prostate cells, Kreb's cycle is utilised, causing an increase in glucose utilisation favourable for the growth of prostate cancer.

Many types of cancer exhibit a phenomenon known as the Warburg effect, where cells undergo rapid aerobic glycolysis instead of oxidative phosphorylation to increase the glucose uptake into the cells, allowing enough energy to be produced for uncontrolled cell growth leading to cancer formation. However, this cannot be the case in prostate cancers, as the prostate already undergoes aerobic glycolysis in healthy cells. The Warburg effect is only exhibited in this cancer's later stages, such as castration‐resistant prostate cancer (CRPC) [184]. A study done by Dyshlovoy et al. [191] showed them modifying naturally found 1,4‐naphthoquinones through conjugation with a glucose molecule (Figure 12). These glucose‐conjugated 1,4‐naphthoquinones were then used to target the Warburg effect in CRPC by disrupting the oxidative phosphorylation within the mitochondria. The results from this study show that this compound is highly effective in its function, suppressing cancerous cells' survival mechanisms and allowing current therapies to be effective once again.

White et al. [196] theorised that the androgen receptor's (AR) part in prostate cancer may hold the key to increased glucose utilisation in the prostate. Their study showed that AR signalling in this disease led to *increased SLC2A12 expression (the gene responsible for glucose transporter GLUT12)*. GLUT12 is responsible for glucose intake into the prostate cells. This results in improved performance of Kreb's cycle in prostate cancer cells in AR‐positive cancer types. However, this does not infer that androgen deprivation therapy will be effective, as this has been shown to lead to changes in glucose metabolism in prostate cancer cells, ultimately resulting in increased glycolysis rates and cancer survival after androgen deprivation [197].

#### 8.1.3 Fatty Acid‐Targeted Treatment

Prostate cancer can cause de novo lipogenesis within the prostate cells, a function not associated with healthy prostate cells. This process is responsible for the formation of fatty acids. De novo lipogenesis, commonly known as fatty acid synthesis, is critical to the survival of various cancers, the prostate variety included [198]. Balaban et al. [199] conducted a study using etomoxir to treat prostate cancer patients with irregular fatty acid synthesis. Etomoxir is an anti‐fatty acid oxidation agent, and this study showed that treatment with the drug decreased cell proliferation and, in turn, reduced cancer growth. Etomoxir works by inhibiting the enzyme CPT1a (Carnitine Palmitoyl‐Transferase1A), whose function is to catalyse a long‐chain acyl group transfer to carnitine from acyl‐CoA, which ultimately results in the oxidation of fatty acids [200]. The chemical structure of etomoxir can be seen in the figure below (Figure 12).

One reason fatty acid synthesis is activated in cancer cells is that it increases the chances of survival and growth within the cancer [198]. This pathway leads to more aggressive forms of cancer, which leads to drug resistance to commonly used therapeutics. Lounis et al. [198] conducted a study where they found that fatty acid synthesis led to resistance to the drug enzalutamide, which targets the AR. However, when combining enzalutamide with a fatty acid synthesis inhibitor such as Stearoyl CoA Desaturase 1 (SCD1), the effectiveness of the two combined proved to be better than when using each drug separately, implying that these two drugs combined can be an effective treatment method. The chemical structure of enzalutamide is shown in the figure below (Figure 12).

#### 8.1.4 Glutamine‐Targeted Treatment

Another common substrate aggressive forms of prostate cancer rely on is glutamine for optimal metabolism in cancer cells. Glutamine catabolism, known as glutaminolysis, acts as a balance regulator for nitrogen and carbon within Kreb's cycle by converting glutamine into the intermediate α‐ketoglutarate [201]. This study showed that glutamine transporters such as SLC1A4 and 5 are often overexpressed in this disease, while their expressions are often correlated to the activity of the AR. This overexpression causes glutamine levels to be high enough to sustain the uncontrolled cell growth associated with prostate cancer [202]. Around half of all prostate cancer patients who undergo radiotherapy will develop recurrence after treatment, with the recurring cancer being more resistant to current therapies [203]. However, Thiruvalluvan, Billet and Bhowmick [202] showed that L‐asparaginase (L‐ASP), commonly used in leukaemia treatments, effectively depletes excess glutamine within the cancer cells, increasing sensitisation to radiotherapies.

### 8.2 Inflammation‐Based Treatment Methods

Inflammation has long been associated with many different cancers. In prostate cancer, specifically, certain environmental factors exacerbate inflammation of the organ, which can act as a catalyst for cancer growth [204]. These environmental factors include obesity, diet and each individual's microbiome, each of which contributes to an increased risk of chronic inflammation. However, inflammation is often described as a symptom of cancer, not a cause itself. Platz et al. [205] set out to determine whether inflammation could be a cause of prostate cancer and not simply a symptom. To achieve this, they had to prove that prostate inflammation preceded the cancer. Their results proved their hypothesis correct, indicating that benign tissue inflammation positively correlates to the onset of cancer.

Cancer development in inflammation‐associated cancers is complicated. Inflammation in the prostate results in reactive oxygen species levels increasing, resulting in damage to DNA in prostate cells [206]. If continued, this build‐up of DNA damage can result in tumour suppressor gene function loss, leading to increased cancer risk [204]. Building on this, inflamed prostate cells typically secrete chemokines and cytokines, which promote cancer growth and metastasis [207]. Thus, it is possible that anti‐inflammatory agents could be used as a form of cancer treatment for inflammation‐associated cancers.

**FIGURE 13:**
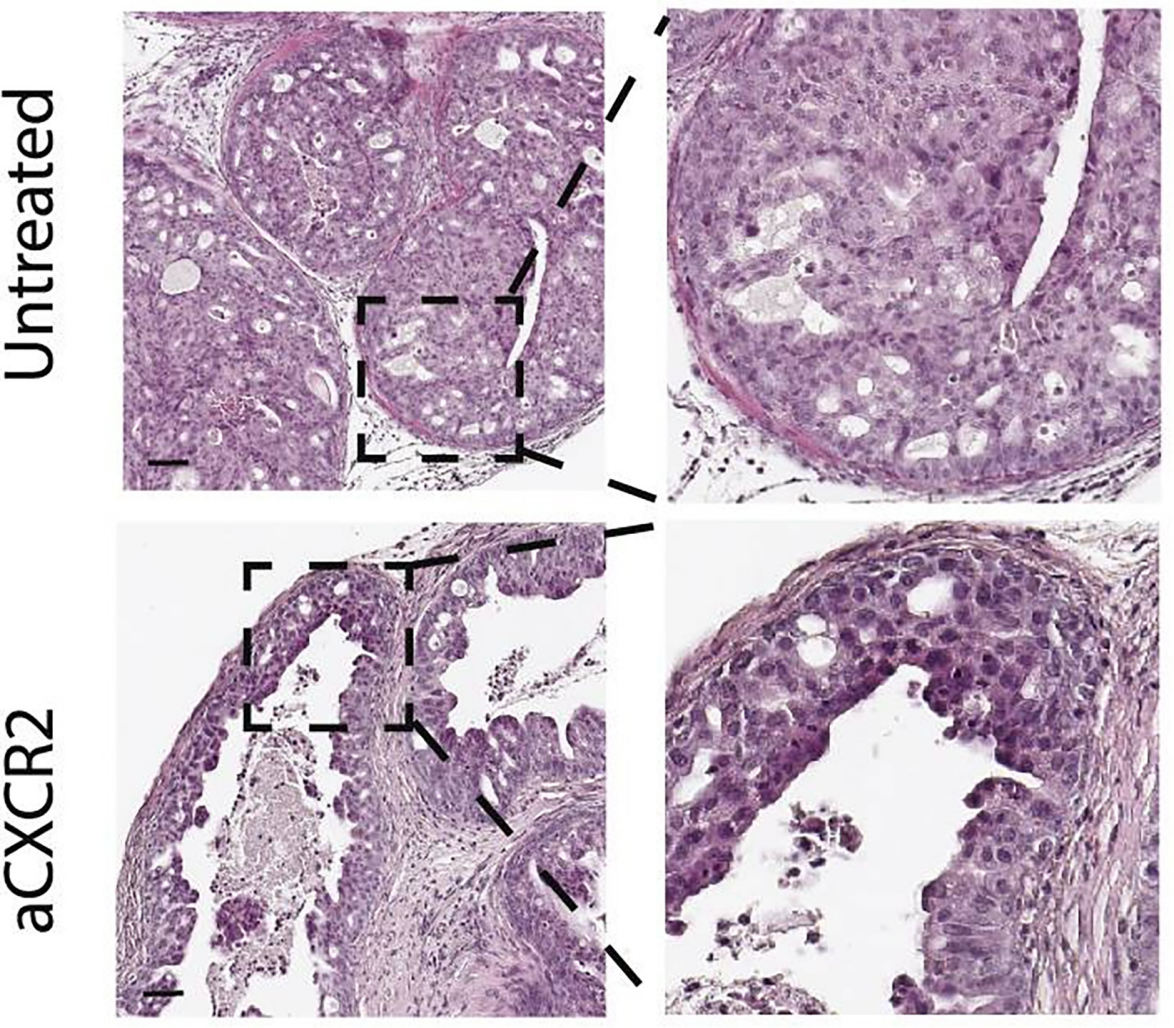
Haematoxylin and Eosin (HE) stain images of prostate tumours before and after treatment with AZ5069, showing tumour suppression and increased normalised prostate cells after treatment. *Image Source:* [212] (Open License CC BY‐NC‐ND).

One of the most prominent promoters of inflammation‐based cancers is tumour‐associated macrophages (TAMs), which are heavily linked to less‐than‐ideal prognoses [208]. TAMs encourage cancer progression by creating genetic instability through cell proliferation and angiogenesis, along with blocking adaptive immune responses [209, 210, 211]. If these macrophages were to be re‐educated from TAMs back to anti‐tumorigenic macrophages, this would have the potential to be an effective cancer treatment. Di Mitri et al. [212] conducted a study that found prostate tumours where *PTEN* expression was inhibited were more susceptible to TAM infiltration. The TAMs in these tumours are highly expressive of the CXC chemokine receptor type 2 (CXCR2), which drives TAM polarisation toward tumour progression. Activation of this receptor is achieved through CXCL2. Blocking this activation is assumed to inhibit the tumour growth and re‐education of the TAMs. Di Mitri et al. [212] proved this possible with the use of the CXCR2 antagonist AZD5069 in treating prostate cancer, where using the antagonist caused a block of tumorigenesis through TAM re‐education into pro‐senescent phenotypes (Figure 13).

**FIGURE 14:**
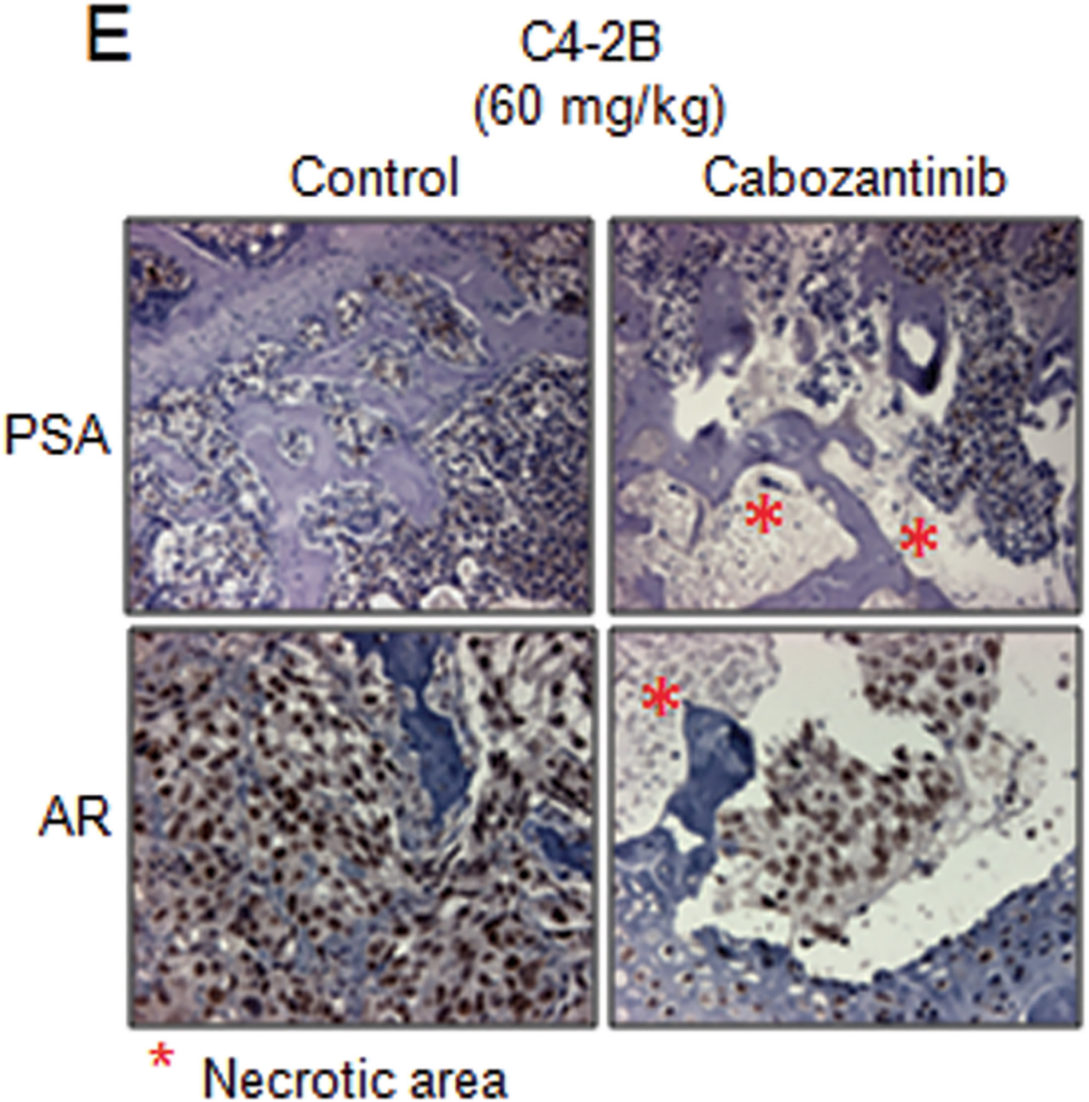
Cabozantinib treatment results in decreased AR and PSA immunoreactivity, with necrotic areas in the tumours after treatment. *Image Source:* [219] (Open License CC BY).

Another immune cell associated with early‐stage inflammation is the neutrophil [213]. Typically, neutrophils have a short lifespan and act in response to bacterial infections [214]. However, in cancer cases, tumour cells have been known to secrete cytokines, extending the neutrophils' lifespan [215]. Chemokines are also secreted by the tumour cells, which lure the neutrophils into the cancerous environment, transforming them into tumour‐associated neutrophils (TANs) [216]. TANs promote cancer progression through immunosuppression, with high neutrophil‐lymphocyte ratios often associated with high Gleason scores in early prostate cancer, leading to poor prognoses [215, 217]. However, neutrophils are also related to the repression of tumour growth. So Patnaik et al. [218] conducted a murine model study where they used cabozantinib, a tyrosine kinase inhibitor, to treat *PTEN/p53* deficient prostate cancers, where the results showed high levels of tumour regression due to large‐scale neutrophil invasion into the tumours. Nguyen et al. [219] conducted a study where cabozantinib treatment on a C4‐2B prostate cell model resulted in decreased AR and PSA immunoreactivity and reduced growth of prostate cancer (Figure 14).

**FIGURE 15:**
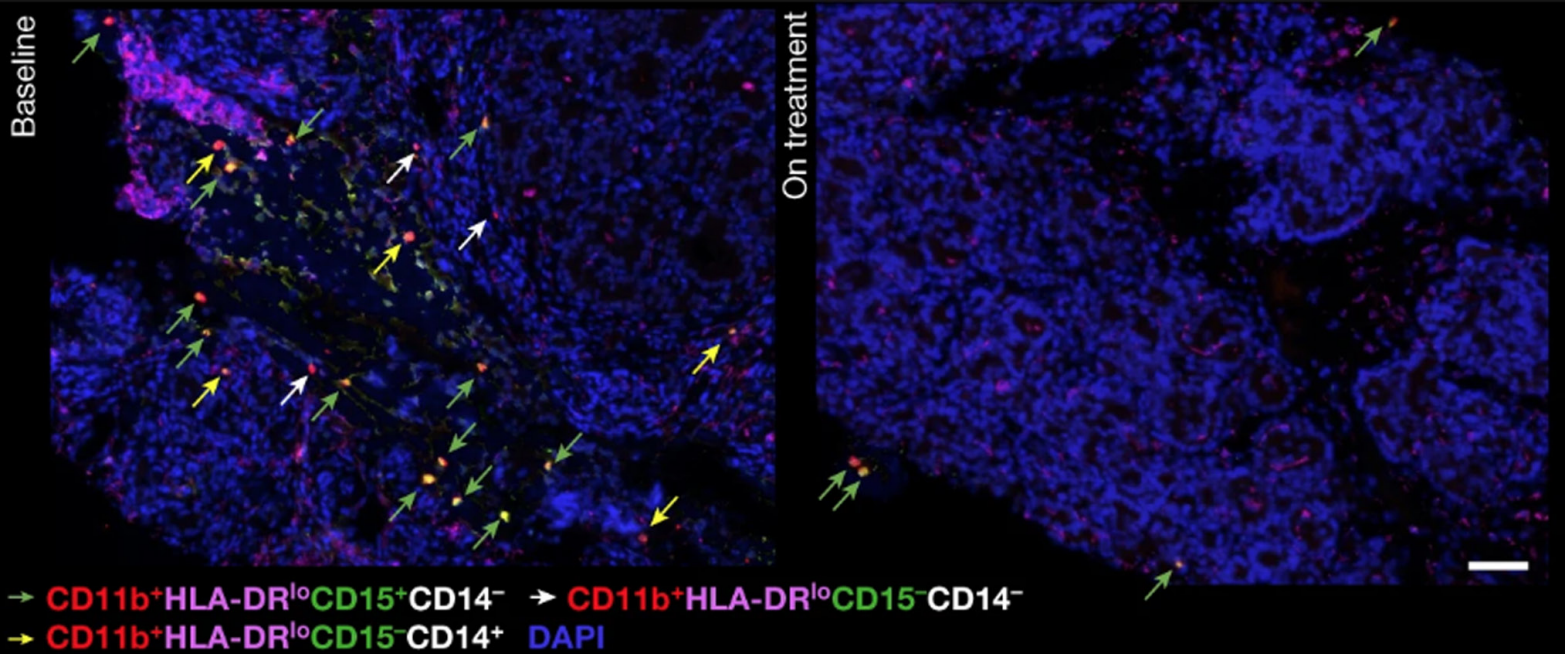
Prostate Cancer biopsy images showing the difference in myeloid cell counts before and after treatment with AZD5069 and enzalutamide. Scale bar = 100 μm. *Image Source:* [226] (Open License CC BY).

A third inflammation‐associated cell type involved in prostate cancer progression is myeloid‐derived suppressor cells (MDSC). They originate in the myeloid tissue and are highly heterogeneous in nature, with the ability to suppress immune responses [220]. These suppressive abilities include inhibition of T cells and natural killer cells, along with suppressing dendritic cell maturation [221, 222, 223]. Calcinotto et al. [224] identified MDSCs to have castration‐resistant effects, as these cells may activate the androgen receptor pathway during androgen deprivation therapy, leading to cancer survival and growth. MDSCs T cell infiltration inhibition into the prostate tumour, therapies which target MDSC inhibition or depletion could be effective strategies against prostate cancer [225]. Chemokines recruit MDSCs during prostate cancer, and so therapies which target chemokine receptors, like the aforementioned CXCR2, could provide more treatment options. Guo et al. [226] conducted a clinical trial where AZD5069 was used in conjunction with the AR antagonist enzalutamide, which reduced MDSC infiltration in metastatic prostate cancer, showing the efficacy of this therapy (Figure 15).

### 8.3 Cytochrome P450‐Based Treatment Methods

The Cytochrome P450 (CYP) family is made up of 57 different genes which encode enzymes in humans that are responsible for the hydroxylation of compounds through the use of oxygen and NADPH (Nicotinamide adenine dinucleotide phosphate) [227]. Several *CYP* enzymes are associated with the metabolism of different lipids, including fatty acids and cholesterol, while others are involved in the oxidation that leads to eventual molecule metabolism of those like vitamin D and androgens [228, 229]. If these *CYP*‐dependent processes were to be disrupted, the consequent outcomes could lead to cancer formation or progression.

**FIGURE 16:**
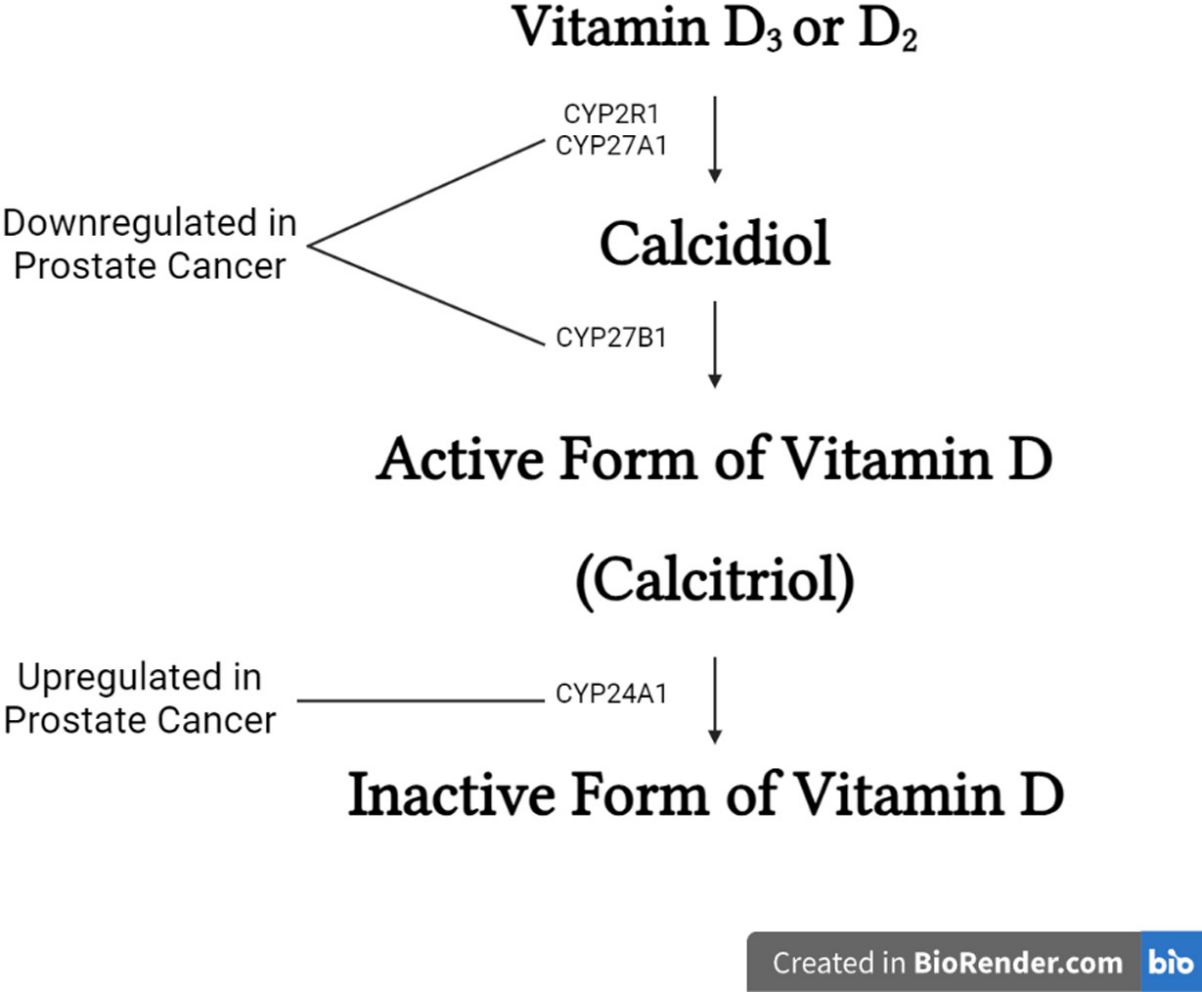
Diagram showing the different CYP enzymes involved in prostate cancer. CYP2R1, CYP27A1 and CYP27B1 are all downregulated in prostate cancer, while CYP24A1 is upregulated. Image created using www.BioRender.com (2024).

CYP2R1 is a CYP enzyme involved in vitamin D activation. Specifically, this enzyme exhibits vitamin D hydroxylase activity, where it converts pro‐vitamin D into calcidiol [230, 231]. This calcidiol has been shown to bind to vitamin D receptors and act as an agonist, modulating target gene expression resulting in antitumour activity [230]. Because of this, Chiang and Chen [232] hypothesised that decreased CYP2R1 expression leading to reduced calcidiol levels could result in an increased risk of cancer development. Down‐regulation of other CYP enzymes such as CYP27A1 and CYP27B1, as well as upregulation of CYP24A1 is linked with prostate cancer development [233, 234, 235] (Figure 16).

**FIGURE 17:**
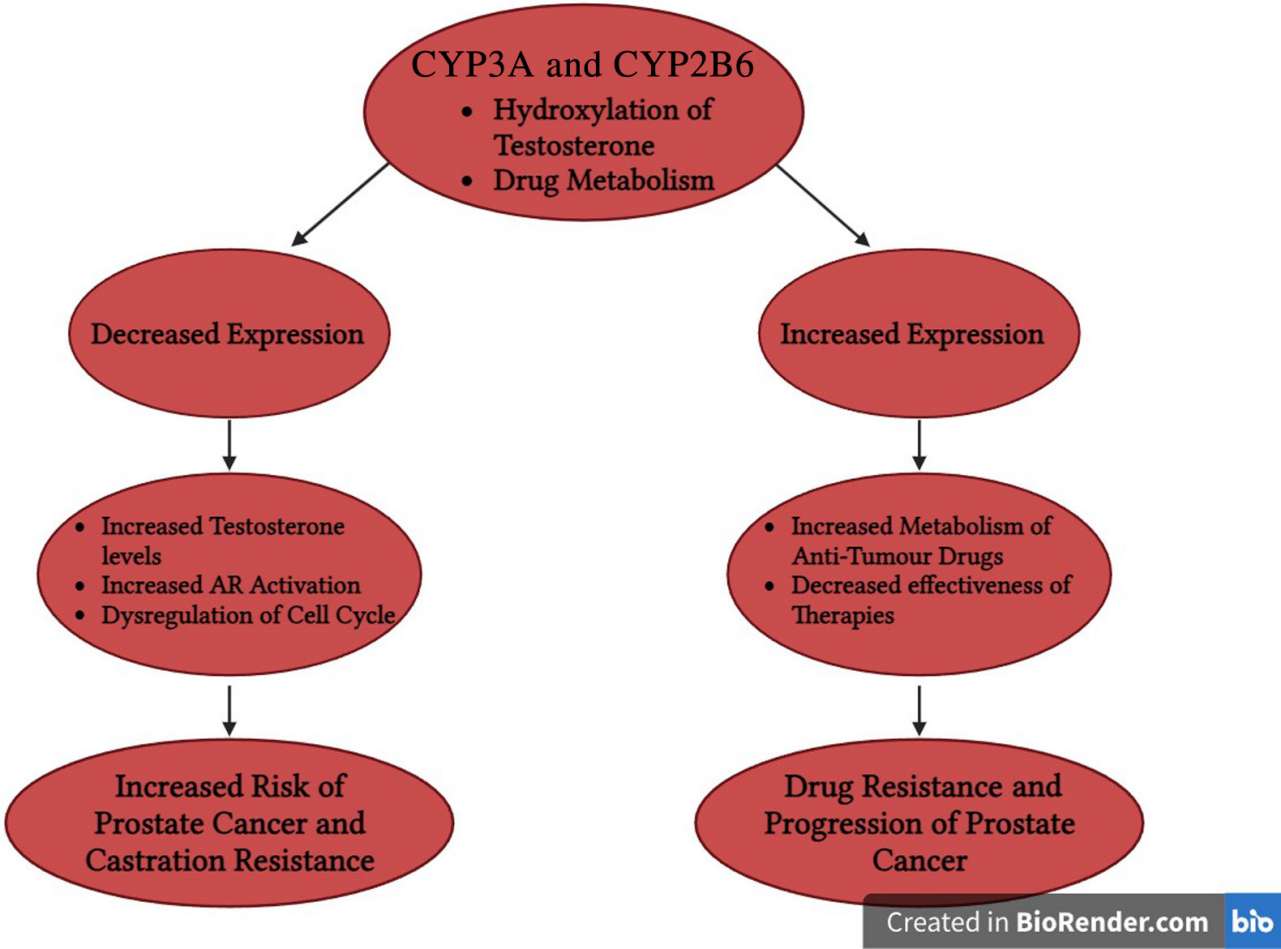
Diagram showing the effects of increased and decreased expression of the CYP3A and CYP2B6 enzymes in prostate cancer progression. Image created using BioRender (www.BioRender.com). Image Information: [230].

CYP3A and CYP2B6 are both CYP enzymes associated with androgen metabolism. As previously alluded to, the androgen receptor (AR) plays a fundamental role in this disease's development and progression. The two enzymes mentioned above are both associated with testosterone metabolism. Specifically, the hydroxylation of testosterone, reducing its activity [236, 237]. Testosterone inactivation leads to inhibiting AR signalling. If the two enzymes' expressions were reduced, this would result in higher testosterone levels, which causes higher AR activity levels and eventual cell dysregulation, ultimately leading to heightened prostate cancer risk and castration resistance. These enzymes are monooxygenases, which allows their functionality in the metabolism of anti‐tumour drugs [230]. Therefore, increased drug metabolism could occur if these enzymes' expression were upregulated, leading to less effective therapies and resistance to these treatments (Figure 17).

The levels of testosterone in circulation also depend on its biosynthesis, which is managed by steroidogenic enzymes [238]. Some of these steroidogenic enzymes include CYP17A1 and CYP11A1, which function in testosterone's precursor's biosynthesis, where they work in tandem with one another [239, 240]. Initially, CYP11A1 convert cholesterol into pregnenolone and iso‐caproic aldehyde within the mitochondria. Then, the pregnenolone moves to the cytoplasm, where it meets CYP17A1. This enzyme then converts pregnenolone into 17α‐Hydroxypregnenolone and Dehydroepiandrosterone, a sex hormone precursor [241]. Polymorphisms in these two enzymes can result in a heightened risk of progression to castration resistance [242]. Several studies over the years have shown that the use of inhibitors of these enzymes causes a reduction in testosterone production, which increases the effectiveness of cancer treatments performed in conjunction [243, 244, 245, 246].

## 9 Programs and Software Available for In Silico Biomarker Design

Over the years, many programs have been developed that help researchers quickly and effectively identify and develop biomarkers for many human and animal diseases. Many web‐based programs are free, with the National Centre for Biotechnology Information (NCBI) having different databases to search and various tools for analysis and comparison (https://www.ncbi.nlm.nih.gov/). Other online tools include UniProt (https://www.uniprot.org/), along with several universities and organisations developing their open‐access bioinformatic tools, including the University of Witwatersrand (available at https://www.wits.ac.za/health/hvdru/bioinformatics‐tools/) and the KwaZulu‐Natal Research Innovation and Sequencing Platform (KRISP, available at https://www.krisp.org.za/tools.php). Along with these online tools, there is also downloadable software readily available for use in in silico analyses, including the Molecular Evolutionary Genetic Analysis (MEGA) software developed by Tamura et al. [247], as well as coding languages that can be used to analyse large quantities of data quickly. Some of the most commonly used coding languages in bioinformatics include Python, Perl and R. Each of these tools mentioned above will be discussed further below.

### 9.1 National Centre for Biotechnology Information (NCBI)

The NCBI is one of the leading open‐access databases within the science community at large, having been developed initially back in 1988 [248]. The NCBI is home to many databases, including those for genes, proteins, literature, chemicals and genomes [249]. The two major databases found on NCBI are GenBank and PubMed. GenBank is an open‐access database for nucleotide sequences, and as of February 2024, was home to more than 25 trillion nucleotide bases from over 3 billion sequences [250, 251]. PubMed is a literature database formed in 1997, with a redesign occurring in 2020 [252]. Although PubMed may have more than 36 million citations and abstracts available, not all of these are full articles, but where possible, links to the free full articles are made available [253].

### 9.2 Universal Protein Resource (UniProt)

UniProt, like NCBI, is home to multiple databases but focuses purely on protein sequences and annotation data [254]. UniProt comprises three databases, including UniProt Archive (UniParc), which contains most protein sequences that are publicly available worldwide. UniParc does not contain duplicate sequences and gives each sequence a unique identifier. The following database is UniProt Knowledgebase (UniProtKB), which includes functional protein information. The last database is UniProt Reference Clusters (UniRef), which allows the user access to sequence sets from the UniProtKB and UniParc to obtain complete coverage of a protein sequence space. UniProt was brought together and created by the UniProt Consortium, a group of three organisations which maintain the service. These three organisations are the Swiss Institute of Bioinformatics (SIB), the Protein Information Resource (PIR) and the European Bioinformatics Institute (EBI).

### 9.3 University of Witwatersrand Bioinformatics Tools

As mentioned, many universities develop their own bioinformatics tools and software to cater to their research needs. These tools could be designed to assist researchers in their specific field and help streamline their work. The University of Witwatersrand's (Wits) Hepatitis Virus Research Unit is one such institute which has developed a line of online bioinformatics tools using sequence data from the Hepatitis B virus. However, these tools can be used for data from any organism, so long as the correct format is used. The tools developed include the Mutation Reporter tool [255], Divergence tool [256] and the Small Genome tools [257].

### 9.4 KRISP (KwaZulu‐Natal Research Innovation and Sequencing Platform)

KRISP is an institute created by the University of KwaZulu‐Natal, the Technology Innovation Agency (TIA) and the South African Medical Research Council (SAMRC). KRISP employs individuals skilled in bioinformatics and computational biology and provides services such as education, support and maintenance of different tools developed by their employees, such as Genome Detective [258] and phylogenetic typing tools [259].

### 9.5 Molecular Evolutionary Genetic Analysis (Mega X)

Mega X is a downloadable software that can be used for several functions, including multiple sequence alignment and evolutionary analysis. Mega X uses a graphical user interface (GUI) that is easy to use and has little to no training. Mega X allows the user to insert sequences and create alignments, compute evolutionary distances and diversities and conduct phylogenetic analyses [260].

### 9.6 Programming Languages Used in Bioinformatics

Over the years, several programming languages have been utilised for bioinformatics, with the rise of big data creating the need for more automated methods in bioinformatics research. As previously alluded, the three most common programming languages used in bioinformatics are Python, R and Perl. Each of these will be discussed further in Table 2.

**TABLE 2:** Different programming languages used in bioinformatics, along with their uses, advantages and disadvantages.

Programming language	Uses	Advantages	Disadvantages	References
Python	Web application scripting, computer communications, artificial intelligence, game development, 2D and 3D imaging, scientific computing	Free, open access, large user base, fastest‐growing language worldwide	Longer codes are more difficult to maintain, a higher level of expertise is needed, slower performance	[261, 262, 263]
Perl	Sequence analysis, general purpose language developed for text manipulation, system administration	Easy to learn, fast script‐writing, BioPerl toolkit allows for even more efficient writing through module re‐use	Older programming language, with fewer uses in bioinformatics than newer languages	[264, 265, 266, 267]
R	Statistical computing, data science, bioinformatics	Large user base, many expansions/libraries available to assist in even more specific tasks, allowing for even more diverse uses	Complicated language, slower than other languages, not as secure as other languages, takes up a more significant amount of memory	[268, 269, 270]
MATLAB	Signal processing, machine learning, control systems, image processing, computational biology and finance	Many toolkits are available for expansion; numerical computation is powerful, and fast performance	Not open access, requires payment to use, slower execution time, requires a large amount of storage and memory	[268]

Once known as the greatest programming language for bioinformatics, PERL has since fallen to the wayside due to the growing popularity of Python and R. Python is the more popular option globally due to its more versatile nature, with its ability to perform tasks outside of the world of statistical analyses and bioinformatics. At the same time, R is utilised mainly for these tasks and has formed its own community.

## 10 Conclusion

Summarising all that has been mentioned above, prostate cancer is among the most critical cancer subtypes worldwide due to its commonality in men, along with its recurrence after initial treatment and resistance to traditional treatment plans. There are many genes associated with this disease's onset and progression, with many of them going unstudied, resulting in an untapped mine of possibilities which could allow for a greater success rate in cancer treatments. Currently, the most common diagnostic and prognostic approaches consist of PSA tests and invasive prostate biopsies, with very few practical alternatives to these tests. Although alternative markers are being studied, there remain significant gaps in research which, if filled, could allow for advancements in our understanding of prostate cancer progression. Therefore, it is of utmost importance that novel biomarkers are identified along with the continued effort to research those currently available markers.

If even a handful of genes were analysed further through mutational analysis and functional effects predictions, this could open a wide array of developments, such as diagnosis and prognosis marker development, identification of personalised treatment plans and ultimately improved patient outcomes. There is hope for a bright future where prostate cancer is not the terrifying disease many people see it to be.

## Author Contributions

**Trevor K. Wilson:** investigation, writing – original draft, validation, writing – review and editing, methodology, data curation, software, formal analysis. **Oliver T. Zishiri:** investigation, funding acquisition, writing – original draft, methodology, validation, writing – review and editing, formal analysis, software, project administration, supervision, resources, data curation.

## Conflicts of Interest

The authors declare no conflicts of interest.

## Data Availability

Data sharing is not applicable to this article as no new data were created or analysed in this study.
